# Mitochondrial impairment in the five-sixth nephrectomy model of chronic renal failure: proteomic approach

**DOI:** 10.1186/1471-2369-14-209

**Published:** 2013-10-04

**Authors:** Larisa V Fedorova, Anita Tamirisa, David J Kennedy, Steven T Haller, Georgy Budnyy, Joseph I Shapiro, Deepak Malhotra

**Affiliations:** 1 Department of Medicine, University of Toledo School of Medicine, Toledo, OH 43614, USA

**Keywords:** 5/6 nephrectomy, Mitochondria, Autophagy, BNIP3, Chronic kidney failure

## Abstract

**Background:**

Kidney injuries provoke considerable adjustment of renal physiology, metabolism, and architecture to nephron loss. Despite remarkable regenerative capacity of the renal tissue, these adaptations often lead to tubular atrophy, interstial and glomerular scaring, and development of chronic kidney disease. The therapeutic strategies for prevention of the transition from acute kidney damage to a chronic condition are limited. The purpose of this study was to elucidate large-scale alterations of the renal cortex proteome in partially nephrecromized rats at an early stage of chronic kidney disease.

**Methods:**

Sprague–Dawley 5/6 nephrectomized rats and sham-operated controls were sacrificed at day 28 post-surgery. To identify proteins with notable alteration of expression we applied a 2D-proteomics approach followed by mass-spectrometry. Altered expression of identified and related proteins was validated by Western blotting and immunohistochemistry.

**Results:**

Proteins with increased levels of expression after partial nephrectomy were albumin and vimentin. Proteins with decreased expression were metabolic or mitochondrial. Western blotting analysis showed that the renal cortex of nephrectomized rats expressed decreased amount (by ~50%) of proteins from the inner mitochondrial compartment - the beta-oxidation enzyme MCAD, the structural protein GRP-75, and the oxidative phosphorylation protein COXIV. Mitochondrial DNA copy number was decreased by 30% in the cortex of PNx rats. In contrast, the levels of an outer mitochondrial membrane protein, VDAC1, remained unchanged in remnant kidneys. Mitochondrial biogenesis was not altered after renal mass ablation as was indicated by unchanged levels of PPARγ and PGC1α proteins. Autophagy related protein Beclin 1 was up-regulated in remnant kidneys, however the level of LC3-II protein was unchanged. BNIP3 protein, which can initiate both mitochondrial autophagy and cell death, was up-regulated considerably in kidneys of nephrecomized rats.

**Conclusions:**

The results of the study demonstrated that notable alterations in the renal cortex of 5/6 nephrectomized rats were associated with mitochondrial damage, however mitochondrial biogenesis and autophagy for replacement of damaged mitochondria were not stimulated. Accumulation of dysfunctional mitochondria after 5/6 nephrectomy may cause multiple adjustments in biosynthetic pathways, energy production, ROS signaling, and activation of pro-cell death regulatory pathways thus contributing to the development of chronic kidney disease.

## Background

Chronic kidney disease (CKD) affects about 10% of patients worldwide [1]. The disease often develops from acute kidney injury if normal renal tissue repair is disrupted or the causes of injury are persistent. Chronic stages of the disease are marked by progressive kidney remodeling and fibrosis which, once is initiated, is very difficult to restrain or reverse. In spite of numerous investigations with various animal models of CKD, the detailed mechanism of tubular atrophy coupled with the increased interstitial scarring remains poorly defined [2].

The partially nephrectomized (PNx) rat is a well-established model for investigation of archetypal pathological changes in CKD. Similar to human disease, the remnant kidney develops adaptive compensatory growth several days after injury. The later stages of CKD in humans and in the rat models are associated with progressive depletion of tubular and glomerular cells. This ultimately leads to glomerular obsolescence, tubular atrophy, and tubulointerstitial fibrosis [3]. In our study, we employed a two-dimensional proteomic analysis of kidney cortex tissue from PNx and sham operated rats to avoid preconceived bias toward any existing hypothesis of CKD progression. We found that the majority of proteins with altered expression in kidneys of PNx rats were related to metabolism. We also demonstrated that metabolic alterations in the PNx kidneys were associated with a significant reduction of mitochondrial DNA and proteins without concomitant changes in mitochondrial biogenesis and autophagy.

## Methods

### Animals

All surgical procedures were conducted in accordance with the National Institute of Health Guide for the Care and Use of Laboratory Animals using protocol approved by the University of Toledo Institutional Animal Use and Care Committee. Two different sets of Male Sprague–Dawley rats (5 sham operated and 9 PNx in each set) weighting ~ 250 g were used for proteomic analysis and western blotting. Five-sixths (5/6) nephrectomy was induced by surgical removal of the right kidney and ligation of two-thirds of the arterial supply to the left kidney with silk ligatures as described previously [4]. Animals were sacrificed at day 28 post surgery.

### Creatinine and creatinine clearance

At the end of the study 24 h urine samples were collected. Blood samples were obtained from abdominal aorta immediately after sacrificing. Creatinine levels were measured calorimetrically with a commercial kit from Teco Diagnostics (Anaheim, CA) as previously described [5].

### Protein extraction

The renal cortical tissues were separated from medulla and ground in liquid N_2_ followed by a sequential extraction of the renal proteins. The frozen protein powder was immediately placed into 40 mM Tris base, containing FOCUS ProteaseArrest protease inhibitors cocktail (G-Biosciences, Maryland Heights, MO), shaken for 30 min at 4°C, and then centrifuged at 15 000 g, 4°C for 30 min. The supernatant was saved as Extract 1. The pellet was then left to dissolve overnight on a shaker at 4°C in 8 M Urea, 4% CHAPS, 40 mM Tris base, 0.2% Bio-Lyte 3/10 (Bio-Rad, Hercules, CA) and 2 mM TCEP as a reducing agent (Pierce, Rockford, IL). The next day, the extraction mixture was centrifuged at 15 000 g, 4°C for 30 min. The collected supernatant was saved as Extract 2. The remaining pellet was dissolved in 5 M Urea, 2 M Thiourea, 2% CHAPS, 2% SB 3–10, 40 mM Tris and 0.2% Bio-Lyte for 1 hour at room temperature following centrifugation at 15 000 g, room temperature for 30 min. The supernatant was saved as Extract 3.

For Western blotting, the frozen protein powder was transferred into RIPA buffer, containing 50 mM Tris–HCl, pH 7.5, 150 mM NaCl, 1% Nonidet P-40, 5% sodium deoxycholate, 0.1% SDS, and Protease Inhibitors Cocktail (Sigma, St-Louis, MO).

Protein concentration was measured by Modified Protein Assay (Bio-Rad).

### Two-dimensional gel electrophoresis and protein identification

17 cm IEF strips with different pI gradients supplied either from Bio-Rad (Bio-Rad) or (GE HealthCare, Piscataway, NJ) were used in the study. To decrease variations between two-dimensional gels we used IEF strips from the same batch to isofocus proteins from control and PNx groups and 2–3 strips for each group were run simultaneously. Protein extracts were diluted in 300 μl of buffer containing 8 M Urea, 2% CHAPS, 0.2% Bio-Lyte 3/10 (Bio-Rad, Hercules, CA), 2 mM TCEP and trace amount of bromphenol blue. The samples were loaded onto the IEF focusing tray, the IEF strip were put on the loaded sample and covered with mineral oil. Isoelectric focusing was run on Protean IEF System (Bio-Rad) for 60,000 V-Hrs. The strips were equilibrated in reduction buffer containing 2% SDS, 50 mM Tris–HCl pH8.8, 6 M urea, 39% glycerol, 0.002% bromphenol blue and 1% of DTT for first 15 min and then in the same buffer but with 2.5% of iodoacetamide instead of DTT for alkylation for 15 more minutes. Before transferring to PAGE the strips were rinsed in the SDS-gel running buffer. Proteins were separated using 5-20% w/v gradient PAGE with piperazine diacrylamine as a crosslinker. The gels were run overnight with the cooling system setting at 10°C. For analytical proteomics gels a total of 50 μg protein were stained with silver, the most sensitive protein stain with low protein to protein variations (Silver Staining Kit, GE HealthCare). Protein spots for identification with mass-spectroscopy were subjectively selected based on visible changes of their amounts on all analytical silver gels (10 gels for every extract of both groups).

Preparative gels were loaded with a total of 500 μg of protein and stained with SYPRO Ruby Stain (Bio-Rad). The level of staining was visually monitored until the selected protein spots were bright enough to cut out. Proteins were in-gel trypsin-digested, extracted, de-salted and send for analysis at the Tufts University Core facility (http://tucf.org). Proteins were identified by SEQUEST search of the rat.fasta database (examples of protein search are shown in Additional file 1).

### Antibodies

The following primary antibodies were used: anti-GRP-75 (1:1000; SPS-825D, Assay Design/Stressgen; Plymouth Meeting, PA), anti-MCAD (1:4000; A21988; Invitrogen, Carlsbad, CA), anti-PGK-1 (1:2000; LS-C81162; LifeSpan BioSciences, Seattle, WA), anti-Beclin 1 (1:1000; 3738; Cell Signaling Technology, Danvers, MA), anti-BNIP3 (1:500; ab-65874; Abcam, Cambridge, MA), anti-LC3 (1:1000, NB100-2220, Novus Biologicals; Littleton, CO), anti-VDAC1 (1:1000; ab15895, Abcam), anti-COXIV (1:1000, ab14744, MitoSciences, Eugene, OR). The following antibodies were purchased from Santa-Cruz Biotechnology (Santa-Cruz, CA): anti-PPARγ (1:500; sc-7273), anti-PGC1α (1:200; sc-5816) and anti-actin (1:1000: sc-1616) and all peroxidase-conjugated secondary antibodies.

### Western blot analysis and immunohistochemistry

Western blotting and immunohistochemistry were performed as described previously [6]. Briefly, equal amounts (10–40 μg) of proteins were loaded onto SDS-PAGE followed transfer to Immobilon-P membrane (Millipore, Billerica, MA) by semi-dry electroblotting. Membranes were then air-dried, rewetted in methanol and blocked in TBS supplemented with 0.1% Tween-20 and 5% non-fat milk or in 5% BSA for Beclin 1 detection. All primary antibodies were used as suggested by the manufacturer. Peroxidase-conjugated secondary antibodies were incubated with the blot for one hour at room temperature and developed using ECL Plus detection reagent (Amersham).

For immunohistochemistry, after deparafinization and rehydration, 5 μm thick kidney sections were boiled in acetic buffer (Vector Laboratories, Burlingame, CA, USA) in a conventional microwave. The endogenous peroxidase activity was blocked with 3% H_2_O_2_ in methanol. The slides were then washed in TBS, blocked in TBS containing 1.5% goat serum for 45 min at room temperature, and probed with anti-MCAD or anti-GRP75 antibodies diluted 1:50 or 1:100 respectively in 1.5% goat serum/BSA in TBS for one hour at room temperature. Finally, the tissue sections were treated according to standard ABC protocol (Vector Laboratories).

### StaRT-PCR for analysis of mitochondrial and nuclear DNA content

Mitochondrial DNA (mtDNA) content was quantified by competitive PCR according to the method described in detail by Willey et al. [7]. Briefly, DNA from whole kidneys was extracted using DNeasy Tissue Kit (Qiagen, Germantown, MD). DNA was amplified in an Eppendorff Thermal Cycler for 35 cycles with primers for mitochondrial genes – cytochrome B (F: TAA ACT CCG ACG CAG ACA AA, R: 5′: GGT GAT TGG GCG GAA TG) and COXII (F: 5′ GCC CTT CCC TCC CTA C, R: 5′ GAC GTC TTC GGA TGA GAT TA), and actin (R: 5′GAG CGG ACA CTG GCA AAG, F: 5′ CAA AGA CCC ATA GGC CAT CA) for nuclear genes. Competitive templates (CT) were: for cytochrom B – 5′CAA AGA CCC ATA GGC CAT CAA CAG ATG CGG CTT AAC ACC C; for COXII -5′GAC GTC TTC GGA TGA GAT TAG GTT TTA GGT CAT TGG TTG G, and for actin – 5′CAA AGA CCC ATA GGC CAT CAA CAG ATG CGG CTT AAC ACC C. 25 μl of PCR reaction contained 20 ng of total DNA, 12.5 μl of PCR master mix (Promega, Madison, WI), 2 μl of each primers and 1 μ of CT mixture containing 10^-15^ moles of mitochondrial templates and 10^-11^ moles of nuclear templates. Reaction products were analyzed on Agilent 2100 Bioanalyser Microfluidic CE Device using DNA 1000 LabChip kit (Agilent Technology, Santa Clara, CA). The native template to competitive template ratio was calculated and the number of mitochondrial or nuclear DNA molecules was estimated according to Willey et al. [7].

### Statistical analysis

Data are presented as the mean ± standard error of the mean. Unpaired Student’s t-test was used to evaluate the difference between two groups. Statistical significance was reported at the *p < 0.05 and **p < 0.01 levels.

## Results

### Analysis of 2D proteomic data

**Figure 1:**
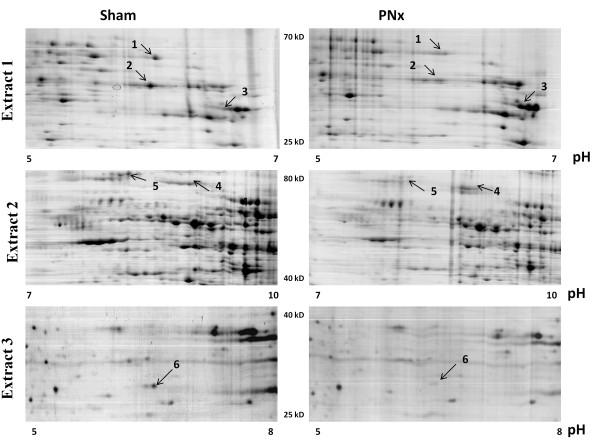
**Differentially expressed proteins from renal cortex of sham operated and PNx rats.** Representative images of 1 from 10 two-dimensional gels run for every protein extract from the control and PNx groups. 2-D gels were stained with Silver. **A** – in cytosolyc fraction **(Extract 1)** the most prominent spots with decreased levels in cortex of PNx rats were identified as MMSDH in spot 1, and PGK1, MCAD and aginonosuccunate synthase 1 in the spot 2. Protein in spot 3 was identified as 40 kD fragment of fibrosis marker protein vimentin. **B** – in hydrophobic fraction **(Extract 2)** the most prominent difference between proteins from PNx and sham operated rats were seen in spots 4 and 5. These proteins were identified as albumin (a marker of tubulointerstitial injury) and GRP-75 (a mitochondrial chaperone molecule) respectively. **C** – in the fraction of membrane-bound proteins **(Extract 3)** of renal cortex, the protein from spot 6 was identified as gamma subunit of ATP-1 synthase.

Physiological measurements of sham-operated and PNx rats are summarized in Table 1. Two-dimensional PAGE revealed in total more than 3000 detectable protein spots from three sequential protein extracts of the renal cortex (Figure 1). The six most prominent spots showing consistent differences between sham-operated and PNx samples were in-gel trypsin digested and identified with matrix–assisted, laser desorption/ionization time-of-flight mass spectroscopy (MALDI-TOF). This analysis revealed eight non-redundant proteins with a high level of confidence based on percentage of protein coverage, peptide number and specific parameters used for validation of SEQUEST search (Table 2). Two proteins with increased level of expression in PNx rats were vimentin and albumin (spots 3 and 4 on Figure 1). Vimentin is a cytosketetal protein which, when expressed, is associated with a mesenchymal phenotype. Immunohistochemical examination of renal sections stained with antibody against vimentin confirmed an increase in vimentin expression in glomerular, interstitial cells, and in arterioles of PNx rats at 28 days post-surgery as previously reported (Additional file 2: Figure S1) [8]. Increased albumin accumulation in proximal tubules and glomeruli of 5/6 nephrectomized rats has also been reported at this time point [9]. One of the proteins identified in spot 2, argininosuccinate synthase, was reported as unchanged [10] or decreased [11] in the same PNx model. Remarkably, the five remaining proteins with decreased levels of expression are, in different ways, involved in energy metabolism and mitochondrial functions.

**Table 1:** Physiological measurements of control and partially nephrectomized rats

	**Sham (n = 10)**	**PNx (n = 18)**
*Body weight (g)*	*458 ± 10*	*440 ± 9*
*Kidney weight (g)*	*1.74 ± 0.09*	2.52 ± 0.17**
*Plasma creatinine (mg/dL)*	*0.32 ± 0.05*	1.16 ± 0.11**
*Creatinine clearance (ml/min)*	*6.12 ± 0.45*	1.06 ± 0.05**
*Hematocrit (%)*	*45 ± 0.9*	39 ± 1*

Values represent group mean ± s.e.m. *p < 0.05 and **p < 0.01 vs control group.

**Table 2:** Renal proteins identified by MALD-TOF mass spectrometry and some parameters used to evaluate SEQUEST search results

**Spot number**	**Protein name**	**Accession number**	**Molecular weight**	**Protein coverage by amino /acid mass (%)**	**Peptide count**	**XCorr value ± SD**	**Delta correlation value ± SD**	**Rank /Sp ± SD**	**Ions (%) ± SD**	**Localization/function**
1	Methylmalonate Semialdehyde dehydrogenase	giI13591997	57770	56.0/56.2	13	3.63 ± 0.93	0.45 ± 0.11	1.23 ± 1.09	67.26 ± 16.52	Mitochondria/ TCA, valine and pyrimidine metabolism
2	Phosphoglycerate kinase 1	giI1730519	44534	44.6/45.2	15	3.36 ± 1.12	0.32 ± 0.16	1.66 ± 2.09	59.31 ± 17.60	Cytoplasm/glycolysis
2	Acyl-coenzyme-A-dehydrogenase (medium chain)	giI8392833	46535	39.0/39.0	13	3.44 ± 1.03	0.37 ± 0.14	1.07 ± 0.27	66.61 ± 9.3	Mitochondria/ β-oxidation
2	Arginosuccinate synthetase 1	giI25453414	46467	55.1/55.7	24	3.80 ± 1.22	0.37 ± 0.15	1 ± 0	65.80 ± 21.33	Cytoplasm/urea cycle
3	Vimentin (fragment)	giI14389299	53697	50.1/50.0	23	3.53 ± 1.18	0.34 ± 0.11	1.26 ± 1.05	76.11 ± 18.50	Cytoplasm/cytoskeleton
4	Albumin (rat)	giI19705431	68674	73.8/73.0	45	3.84 ± 0.79	0.40 ± 0.11	1.06 ± 0.33	71.63 ± 14.14	Plasma/ protein transport
5	Glucose regulated protein 75	giI2119726	73699	53.2/53.1	31	3.97 ± 0.71	0.44 ± 0.11	1 ± 0	72.68 ± 11.90	Mitochondria/chaperon, structural
6	Gamma subunit ATP-1 synthase	giI6729936	29933	36.7/35.9	8	3.49 ± 0.72	0.47 ± 0.10	1.12 ± 0.35	49.42 ± 24.24	Mitochondria/oxidative phosphorylation

### Down-regulation of mitochondrial proteins and DNA in PNx kidneys

**Figure 2:**
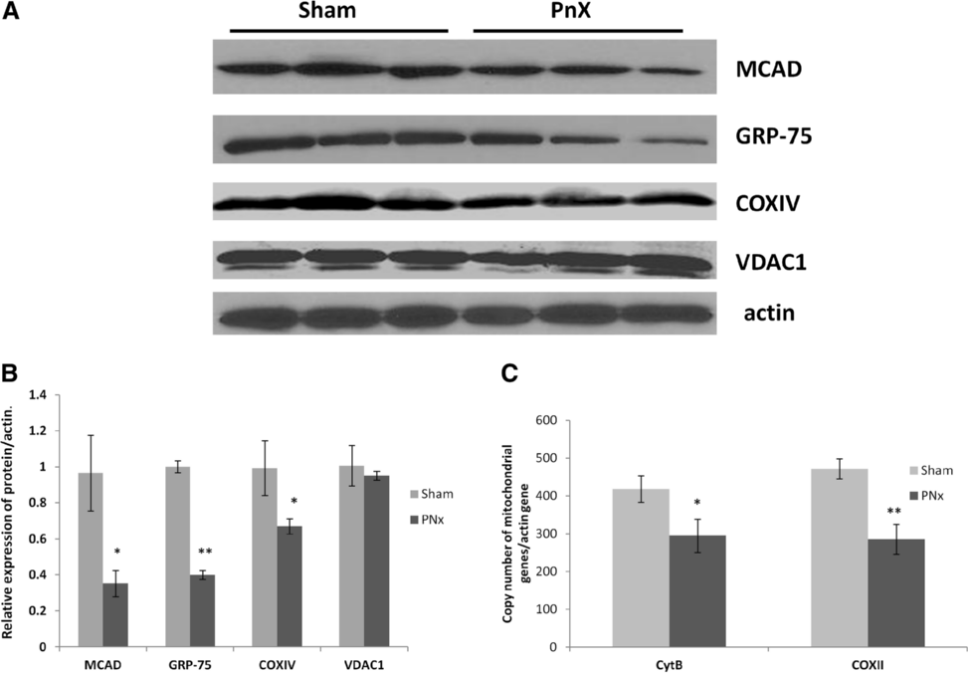
**Decrease of mitochondrial proteins and DNA in the rat kidneys after PNx surgery.** Representative Western blots **(A)** and densitometry analysis **(B)** shows down regulation of MCAD (n = 6), COXIV (n = 6) and GRP-75 (n = 11) in PNx rats when compared to the sham operated controls. *p < 0.05, **p < 0.01. **C** - Sham operated control cortex contains approx. 450 copies of mitochondrial genes COX II and Cytochrom B per nuclear actin gene. Renal mass ablation PNx results in a decrease of mitochondrial DNA below 300 copies per actin. n ≥ 15, *p < 0.05, **p < 0.01.

Three out of the five identified metabolism-related proteins - medium chain acetyl dehydrogenase (MCAD), phosphoglycerate kinase 1 (PGK-1), and glucose regulated protein-75 (GRP-75) - were used for proteomic data validation by Western blotting analysis. The difference in total amounts of PGK-1 protein was not significant between the two groups (data not shown). We used different sets of animals for proteomic and western blot studies so lack of difference in PGK-1 levels between control and PNx kidneys may be due to subtle difference in experimental conditions (such as feeding, time of sacrifice). However, the total levels of MCAD and GRP-75 were significantly decreased (*p < 0.05* and *p < 0.01)* in the renal cortex of PNx rats compared to the sham operated controls in accord with 2D PAGE findings (Figure 2A,B). Since we were not able to find reliable antibodies against γ-subunit of ATP-1 synthase, we analyzed expression of another protein from the oxidative phosphorylation complex, cytochrome c oxidase subunit IV (COXIV). Western blotting demonstrated that COXIV levels were indeed significantly (*p < 0.05)* reduced in PNx kidneys (Figure 2A,B). All three down-regulated proteins, MCAD, GRP-75 and COXIV, are located exclusively in the inner mitochondrial compartment. In contrast, the amounts of the outer membrane protein VDAC1 (voltage-dependent anion channel 1) in the PNx kidneys were similar to those in the sham-operated kidneys.

To further elucidate mitochondrial damage in PNx kidneys we examined mitochondrial DNA content. The renal cortex of sham operated rats contained around 450 copies of mitochondrial genes cytochrome c oxidase subunit II and cytochrome b per nuclear actin gene (Figure 2C). In the cortex of PNx rats the copy number of mitochondrial genes was reduced to around 300 copies per actin gene.

### Immunohistochemical examination of mitochondrial injury

**Figure 3:**
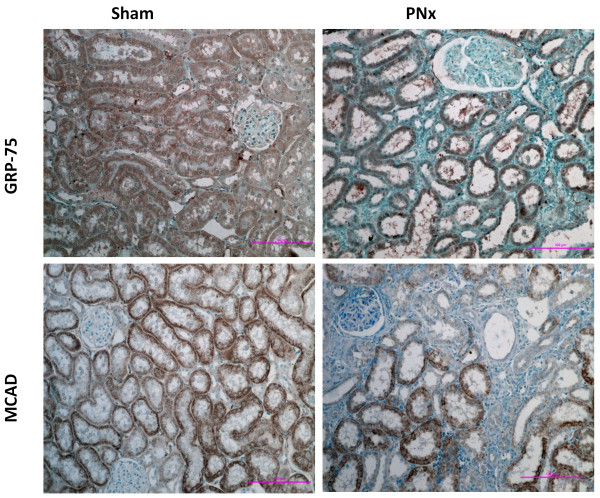
**Representative microphotographs of immunohistochemical examination of renal cortical sections from sham operated and PNx rats.** In sham-operated control kidneys the specific staining for both mitochondrial proteins – MCAD and GRP-75 - is distributed evenly throughout the cortical tissue. After PNx, many tubules (especially in fibrotic and inflamed lesions) showed a marked decrease in expression of mitochondrial MCAD and GRP-75 proteins. Bar = 100 μm. The images were taken on a Nikon Eclipse 80i microscope equipped with a Nikon camera HeadDS-Fi1 (Nikon, Tokyo, Japan).

Mitochondrial injury after renal mass ablation was further examined by immunohistochemistry (Figure 3). In sham-operated rats an intensive specific staining with both anti-MCAD and anti-GRP-75 antibodies was clearly visible in the tubular compartment of the cortex reflecting high mitochondrial density in tubular epithelial cells. In glomerular cells, the levels of MCAD and GRP-75 proteins were below the detectable threshold. Microphotographs of immunohistochemical staining of the cortical sections from PNx rats showed that epithelial cells in some tubules contain high levels of MCAD and GRP-75 while in flattened epithelial cells abundance of these proteins was notably decreased. Therefore, mitochondrial injury was not evenly distributed throughout the cortical tissue in PNx kidenys. Tubules with low mitochondrial density were associated with areas of interstitial inflammation and fibrosis (Figure 3).

### Mitochondrial biogenesis and autophagy after PNx

**Figure 4:**
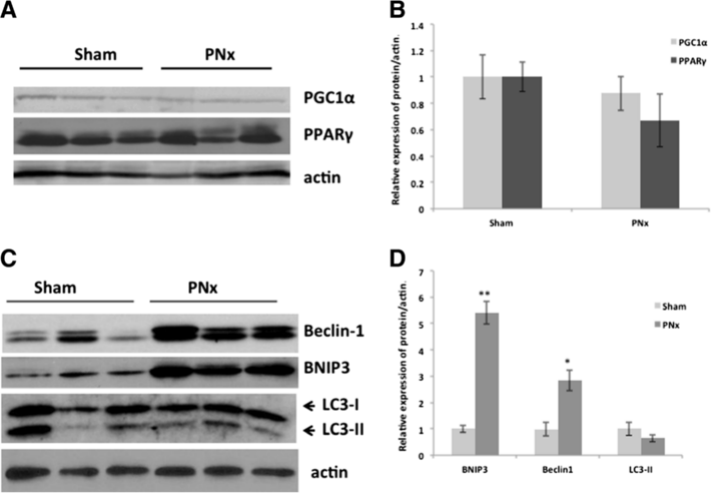
**Mitochondrial biogenensis and autophagy in the cortical tissue of PNx rats.** Representative immunoblot **(A)** and densitometry analysis **(B)** of expression of proteins controlling mitochondrial biogenesis – PGC1α and PPARγ **(A,B)**. No significant changes were found. *n = 10*. Representative western blots **(C)** and densitometry analysis **(D)** of Beclin 1 and BNIP3 demonstrate significant up-regulation of these proteins in PNx kidneys when compared with sham-operated controls. Note two forms of LC3: slowly moving cytoplasmic LC3-I, and more mobile lipidated form of LC3-II **(A)**. *n = 8*, *p < 0.05.

Mitochondrial abundance is determined by a balance between their synthesis and degradation. As shown by Western blotting, the expression levels of two essential molecules of mitochondrial biogenesis, PPARγ and its co-activator PGC-1α [12] were not significantly altered in the cortex of PNx rats (Figure 4A,B).

Damaged mitochondria are eliminated by a specific type of selective mitochondrial autophagy [13,14]. This process is initiated by formation of a double-membrane organelle known as the autophagosome. The Beclin 1 protein is essential for early stages of autophagosome membrane formation [15]. BCL2/adenovirus E1B 19 kDa interating protein 3 (BNIP3) is involved specifically in mitochondrial autophagy either by induction of Beclin 1 levels or by a specific targeting of damaged mitochondria to the autophagosome [16-18]. Both Beclin 1 and BNIP3 proteins were significantly up-regulated *(p < 0.05)* in the PNx kidneys when compared to the sham-operated controls (Figure 4A,B). Later stages of autophagy - autophagosome maturation and closure - are critically dependent on the lipid-conjugated form of LC3 (microtubule-associated protein 1 light chain 3) protein [19]. Both cytoplasmic (LC3-I) and autophagosome-bound lipidated (LC3-II) forms were present in kidneys of sham-operated and PNx rats (Figure 4C). Although the LC3-II levels were decreased in PNx kidneys this difference was not statistically significant (Figure 4D). SQSTM1/sequristome/p62 protein or p62, an ubiquitin-binding protein, is a selective substrate of autophagy and an accumulation of both p62 and ubiquinated proteins serves as an indicator of autophagy inhibition [20-22]. p62 band was not detected by Western blot with exception of one animal which also had a slight increase in abundance of ubiquinated proteins and a very low level of LC3-II (Additional file 3: Figure S2).

## Discussion

### PNx model

Renal mass ablation in PNx rats leads to adaptive hypertrophic growth of the remaining kidney within 7–10 days post-surgery [23]. This growth is accompanied by the development of glomerular sclerosis, hypertensive vascular changes, and tubular atrophy. However, after the fourth week post-surgery, the PNx kidney is characterized by marked retardation of compensatory renal growth, a progressive increase in accumulation of apoptotic cells in every renal cell population, and augmented tubular–interstitial injury and fibrosis [24,25]. We attempted to elucidate major alterations in the cortical proteome associated with progress to the chronic stage of renal disease at that particular stage. Albeit with the inherited limitations of gel proteomics (variations in IEF strips, complexity of protein patterns on the gel, low sensitivity for less abundant regulatory proteins), we identified eight proteins with altered abundance which are associated with three biochemical features of PNx kidneys – *i)* albumin accumulation, *ii)* up-regulation of vimentin reflecting an acquisition of a more mesenchymal phenotype by variety of renal cells, *iii)* and metabolic perturbations. While albumin toxicity and glomerular damage/fibrogenesis have been recently studied in this model [9,26], metabolic alterations specific for PNx kidneys gained much less attention since its first recognition two decades ago [27-30].

Hypertrophic growth of PNx kidneys is accompanied by considerable alterations of the metabolic state of the remaining nephrons (called also hypermetabolism of the remnant kidney) manifested by an increased net glucose production [30], accumulation of inorganic phosphate [28], and inefficient oxygen utilization per nephron and per sodium transport [27-29]. In response to compensatory hypertrophy, all nephron segments become enlarged. However, the proximal tubules grow out of proportion to the rest of the nephron [24,25]. In proximal tubules mitochondria occupy from 22 to 39% (depending of the segment) of the cellular volume [31]. With low capacity for anaerobic glycolysis [32], the adaptation of proximal tubules in remnant kidneys for increased demand in ATP production (needed for electrolyte transport) relies on alterations in the structure and abundance of their mitochondria. Accordingly, at acute stages of renal growth after renal mass ablation (up to 2 weeks) mitochondrial proliferation/hypertrophy has been reported [33,34]. However, early investigations showed that renal mitochondria become uncoupled at 4 weeks post-surgery with trends toward a decrease in ATP production and total oxygen consumption [27,29]. Taken together, these observations are in line with our present data which showed perturbations in renal mitochondria at this stage.

### Mitochondrial damage in PNx kidneys

Mitochondrial proteins and DNA, which we found to be depleted in the kidneys of PNx rats, are located to the inner mitochondrial compartment. GRP-75 is located largely to the inner mitochondrial membrane but also can be found in other sub-cellular sites [35,36]. Considering high mitochondrial density of the renal proximal epithelial cells [31], we assume that GRP-75 protein from the examined cortical extract represents largely its mitochondrial fraction. MCAD, a key enzyme of mitochondrial β-oxidation of fatty acids, and COXIV are both localized exclusively to the mitochondrial matrix and inner mitochondrial membrane respectively. In contrast, the levels of VDAC1 protein, which is ubiquitously situated on the outer mitochondrial membrane did not change in the PNx kidneys

Decrease in mitochondrial proteins abundance in the PNx kidney was distributed heterogeneously throughout the cortex as demonstrated by immunohistochemical analysis of GRP-75 and MCAD (Figure 3). Weak immunostaining for inner mitochondrial proteins was observed only in the flattened epithelial cells of dilated tubules, which were surrounded by enlarged interstitum. Thus, the areas with damaged mitochondria may be associated with poor tubulointerstitial reperfusion and hypoxia as consequences of the distortion and collapse of the peritubular capillaries seen in PNx kidneys from the second week onward [37-39]. The hypoxic milieu stimulates secretion of profibrotic signals from tubular, interstitial and endothelial cells resulting in accumulation of fibrotic tissue [40]. In turn, fibrotic scar formation results in extension of the distance between existing capillaries and tubular cells thus reducing the efficiency of oxygen diffusion. Thus through this cycle of pathological amplification, the portion of ischemic tubules progressively increases [40].

The mitochondrial membrane potential is especially low in proximal tubules and collapses easily under hypoxia [41]. Ischemic insult to renal epithelial cells causes considerable damage to the mitochondrial inner membrane and leads to mitochondrial swelling and disruption of cristae structure [42]. In our parallel investigation of the ischemic 2 Kidneys 1 Clip model of renal stenosis, we also found considerable decrease in abundance of inner mitochondrial proteins with unchanged levels of VDAC1 [43].

### Autophagy in PNx kidneys and in CKD

Damaged mitochondria are a major source of genotoxic oxidant species and pro-death factors. Therefore, mitochondrial quality control is crucial for renal cell survival.

Autophagy, a process by which the entire damaged organelle, including a mitochondrion is eliminated, requires formation of a special double-membrane structure called an autophagosome. The lipidated form of the LC3 protein, LC3-II, which essential for autophagosomal-lysosomal fusion, is a commonly used molecular marker of autophagosome maturation. Total LC-II amount is an indicator of two intracellular activities: *i)* the level of autophagic flux; and *ii)* the rate of LC3-II lysosomal degradation [44,45]. In order to distinguish between these two activities *in vitro*, specific inhibitors of lysosomal proteases are commonly used. However, this method is hard to use *in vivo*[45]*.* Another commonly used marker of autophagy is SQSTM1/sequristome/p62 protein. Yet p62 levels are regulated by several pathways independent of autophagy [46,47]. In PNx extracts the levels of LC3-II isoform was not significantly decreased when compared to controls (Figure 4) and p62 was not detected by Western blot with the exception of one animal which also had a very low level of LC3-II on Western blots (Additional file 3: Figure S2). Thus, the trend to reduction of LC3-II accumulation in combination with unchanged levels of outer mitochondrial membrane protein VDAC1 suggests that autophagy is not likely to be activated in PNx kidneys and thus unlikely to be responsible for reduction in mitochondrial proteins. In the 2 kidneys 1 clip model we did not detect either LC3-II or ATG5 (the protein required for LC3 lipidation) by Western blot yet this model was associated with marked p62 accumulation [43]. Although autophagy stimulation in response to multiple acute kidney injuries has been shown to be protective [48,49], autophagy is seemingly inhibited during prolonged cellular stress and in aged kidney [50-52]. In a cisplatin-induced model of renal deficiency, the autophagic flux is gradually decreased and activation of autophagy during the course of cisplatin toxicity exacerbates kidney injury [53]. Finally, persistently increased autophagy in patients with genetic nephropathic cystinosis has been linked to renal cell injury and apoptosis, and autophagy inhibition has been found to be cytoprotective [54].

Autophagy has been linked with negative regulation of apoptosis [55]. The crosstalk between autophagy and apoptosis is mediated by complex interactions between Bcl-2 proteins. Two members of this family, BH3-only proteins Beclin 1 and BNIP3, were up-regulated in the PNx kidneys (Figure 4). The role of Beclin 1, an important inducer of autophagosome membrane formation, in regulation of apoptosis is not fully understood [56]. Expression of the BNIP3 protein is specifically up-regulated by hypoxia [57,58]. BNIP3 is primarily localized in the mitochondrial membrane [59] but has also recently been found on the endoplasmic reticulum [60]. Under stress condition BNIP3 binds directly to LC3-II through its LC3-interacting region thus delivering target damaged organelles for autophagic degradation [60]. In hypoxic aged kidneys, BNIP3 activation by caloric restriction promotes mitochondrial autophagy and thus enhances kidney adaptation to hypoxia [50]. However, BNIP3 functions are context-dependent and autophagy induction and cell-death promotion are shown to be two separate activities of BNIP3 [17,18,59,61]. As we found in PNx kidneys, autophagy is not likely to be up-regulated, thus BNIP3 autophagic activities may be limited (Figure 4). When autophagy is inhibited, BNIP3 induces swelling of inner mitochondrial membranes and triggers release of pro-apoptotic proteins [62]. Notably, a drastic increase in apoptosis of tubular epithelial begins from 4 weeks after surgery in the PNx models [63]. In addition to apoptosis and autophagy, mitochondria-bound BNIP3 forms a complex with NIX and Mieap proteins, which mediates translocation of lysosomal proteins into the mitochondrial matrix. Through such a mechanism BNIP3 controls elimination of oxidized mitochondrial proteins as shown in cancerous cells [64,65]. Our finding of down-regulation of mitochondrial proteins located in the inner mitochondrial compartments (Figure 2A) suggests that this particular mechanism may be activated by BNIP3 in PNx kidneys. BNIP3 protein involvement in mitochondrial turnover and cell death has been mostly studied in the myocardium [57,59,61,66,67] and in multiple cancer cell lines [17,68,69]. We have recently demonstrated that renal necrosis in the Goldblatt’s 2 kidneys, 1 clip model was associated with up-regulation and activation of BNIP3 protein [43]. Specifically, reduction of oxidative stress by activation of PPARδ signaling resulted in BNIP3 deactivation, preservation of mitochondrial function, and prevention of cell death.

## Conclusion

In conclusion, we found mitochondrial impairment in PNx kidneys and an up-regulation of BNIP3, a key protein in regulation of both mitochondrial quality control and cell death in PNx kidneys. These findings together with our observations in Goldblatt’s 2 kidneys, 1 clip model, suggests that BNIP3 protein may play an important role coordinating the intricate relationship between mitochondrial health, autophagy, and cell-death regulatory pathways in the development of CKD.

## Competing interests

The authors have no conflicts of interest to disclosure.

## Authors’ contributions

Study design: LF, DM and JIS. Study conduct: LF, AT and DK. Data collection: DK, SH and GB. Data analysis and interpretation: LF and JIS. Drafting manuscript: LF. Approving final version of manuscript: LF, AT, DJK, STH, GB, DM, JIS.

## Pre-publication history

The pre-publication history for this paper can be accessed here:

http://www.biomedcentral.com/1471-2369/14/209/prepub

## Supplementary Material

Additional file 1 Sequest summary. Click here for file

Additional file 2: Figure S1 Immunohistochemical analysis of vimentin protein in the cortex of sham-operated and PNx rats. Click here for file

Additional file 3: Figure S2 Representative immunoblot of renal cortical extracts shows accumulation of p62 and very low level of LC3-II in only one PNx rat. There was slight difference in amounts of polyubuquinated proteins in cortex of the same PNx rat. Click here for file
