# Supplementary material for: Mitochondrial impairment in the five-sixth nephrectomy model of chronic renal failure: proteomic approach
Source: BMC Nephrol. 2013 Oct 4;14:209. doi: 10.1186/1471-2369-14-209 (PMC3851543; doi:10.1186/1471-2369-14-209)
Supplement: Additional file 1 — Sequest summary. [file 1471-2369-14-209-S1.pdf]

# Sequest Summary

File | Select | Sequest | Protein | Utilities | Help

**Sample:** Berne, M. (K6) 1 mb
**DataFiles:** K6 (02/27/04-02/27/04)
**Database:** rat (01/05/2004)
**Directory:** mbernek6 View Info
**Diff Mods:** 16 M 57.02 C 0.000 X

**OutFiles:** 1283|944
**Enzyme:** Trypsin_Strict
**Mass:** ±1.5 (Mono)
**Max list:** 1000
**Intensity:** 3.5e9 MS2

DTA VCR

Consensus **Depth:** 3

A gi|13591997|ref|NP_112319.1| 246 40|Σ:9.7e8|28%|avg:2.4e7 {37,3,0,1,1,1}
methylmalonate semialdehyde dehydrogenase gene [Rattus norvegicus] gi|400269|sp|Q02253|MMSA_RAT Methylmalonate-semialdehyde dehydrogenase [acylating], mitochondrial precursor (MMSDH) gi|347034|pir||A44097 methylmalonate-semialdehyde dehydrogenase (acylating) (EC 1.2.1.2

| # | GBU | TIC | File | z | dM | MH+ | xC | dCn | Sp | RSp | Ions | Sf | Ref | () | Sequence | Prob |
|---|---|---|---|---|---|---|---|---|---|---|---|---|---|---|---|---|
| 429 | | 7.9e7 | 1844-1888 | 2 | 0.8 | 1962.4 | 5.90 | 0.59 | 889 | 1 | 22/38 | -- | gi\|13591997 | | (-)VNAGDQPGADLGPLITPQAK | |
| 528 | | 7.3e6 | 2123-2152 | 2 | 0.5 | 1888.6 | 5.59 | 0.59 | 1933 | 1 | 23/32 | -- | gi\|13591997 | | (-)AISFVGSNQAGEYIFER | |
| 422 | | 6.6e7 | 1818-1840 | 2 | 0.6 | 1721.3 | 4.42 | 0.56 | 970 | 1 | 21/28 | -- | gi\|13591997 | | (-)WIDIHNPATNEVVGR | |
| 416 | | 6.9e5 | 1791-1806 | 2 | 0.5 | 2051.7 | 4.32 | 0.44 | 883 | 1 | 18/34 | -- | gi\|13591997 | | (-)SDKWIDIHNPATNEVVGR | |
| 439 | | 2.0e6 | 1893-1898 | 2 | 0.8 | 1962.4 | 4.28 | 0.52 | 772 | 1 | 18/38 | -- | gi\|13591997 | | (-)VNAGDQPGADLGPLITPQAK | |
| 395 | | 2.3e7 | 1754-1778 | 2 | 0.0 | 1465.8 | 4.16 | 0.48 | 1145 | 1 | 19/26 | -- | gi\|13591997 | | (-)C#M*ALSTAVLVGEAK | |
| 428 | | 2.9e6 | 1824 | 3 | -0.9 | 1722.8 | 4.07 | 0.35 | 2031 | 1 | 30/56 | -- | gi\|13591997 | | (-)WIDIHNPATNEVVGR | |
| 294 | | 1.5e7 | 1519-1539 | 2 | -0.3 | 1923.2 | 4.02 | 0.54 | 655 | 1 | 17/34 | -- | gi\|13591997 | | (-)EEDATLSSPAVVM*PTM*GR | |
| 352 | | 1.7e7 | 1649-1680 | 2 | 0.0 | 1194.3 | 3.97 | 0.47 | 1107 | 1 | 17/20 | -- | gi\|13591997 | | (-)TLADAEGDVFR | |
| 482 | | 1.3e6 | 2033-2038 | 2 | -0.6 | 2721.0 | 3.64 | 0.59 | 307 | 1 | 17/48 | -- | gi\|13591997 | | (-)GLQVVEHAC#SVTSLM*LGETM*PSITK | |
| 414 | | 3.1e7 | 1790-1816 | 2 | 0.1 | 1226.3 | 3.34 | 0.33 | 702 | 1 | 15/18 | -- | gi\|13591997 | | (-)QGIQFYTQLK | |
| 555 | | 3.3e6 | 2184-2207 | 2 | 0.3 | 1435.3 | 3.15 | 0.47 | 1025 | 1 | 19/24 | -- | gi\|13591997 | | (-)AFPAWADTSILSR | |
| 299 | | 3.7e7 | 1545-1575 | 2 | 0.4 | 1030.8 | 3.02 | 0.37 | 1253 | 1 | 16/18 | -- | gi\|13591997 | | (-)EGASILLDGR | |
| 288 | | 8.0e7 | 1481-1516 | 2 | -0.4 | 1017.0 | 3.01 | 0.44 | 514 | 1 | 15/18 | -- | gi\|13591997 | | (-)VPGATM*LLAK | |
| 129 | | 1.7e7 | 0750-0801 | 2 | -0.4 | 1197.9 | 2.90 | 0.45 | 724 | 1 | 14/20 | -- | gi\|13591997 | | (-)NHGVVM*PDANK | |
| 248 | | 3.8e7 | 1329-1353 | 2 | -0.2 | 1078.6 | 2.90 | 0.32 | 866 | 1 | 13/14 | -- | gi\|13591997 | | (-)DM*DLYSYR | |
| 356 | | 5.5e5 | 1689-1691 | 2 | 0.6 | 1193.7 | 2.82 | 0.53 | 575 | 1 | 13/20 | -- | gi\|13591997 | | (-)TLADAEGDVFR | |
| 154 | | 5.5e6 | 0855-0883 | 2 | 0.0 | 1166.5 | 2.79 | 0.48 | 1457 | 1 | 16/20 | -- | gi\|13591997 | | (-)AEM*EAAVAAC#K | |
| 351 | | 3.4e7 | 1646-1687 | 1 | 0.4 | 1193.9 | 2.72 | 0.46 | 197 | 1 | 11/20 | -- | gi\|13591997 | | (-)TLADAEGDVFR | |
| 541 | | 5.8e5 | 2168 | 2 | 0.5 | 1888.5 | 2.66 | 0.39 | 482 | 1 | 13/32 | -- | gi\|13591997 | | (-)AISFVGSNQAGEYIFER | |
| 230 | | 3.0e7 | 1235-1267 | 2 | -0.7 | 1077.2 | 2.58 | 0.11 | 583 | 6 | 13/18 | -- | gi\|13591997 | | (-)VC#NLIDSGAK | |
| 176 | | 1.3e6 | 0990 | 2 | 0.0 | 902.0 | 2.27 | 0.35 | 324 | 1 | 11/14 | -- | gi\|13591997 | | (-)GDTNFYGK | |
| 466 | | 2.0e6 | 1961-1968 | 2 | -1.5 | 2693.7 | 2.20 | 0.27 | 175 | 1 | 11/46 | -- | gi\|13591997 | | (-)GYENGNFVGPTIISNVKPSM*TC#YK | |
| 242 | | 3.1e6 | 1296 | 2 | 0.1 | 902.0 | 2.18 | 0.14 | 310 | 2 | 9/14 | -- | gi\|13591997 | | (-)LITLEQGK | |
| 456 | | 1.5e6 | 1925 | 2 | 0.1 | 1042.1 | 2.09 | 0.32 | 368 | 2 | 11/14 | -- | gi\|13591997 | | (-)WLPELVER | |
| 232 | | 1.2e7 | 1236-1270 | 1 | -0.3 | 1076.8 | 2.05 | 0.14 | 625 | 1 | 12/18 | -- | gi\|13591997 | | (-)VC#NLIDSGAK | |
| 240 | | 3.9e7 | 1283-1326 | 1 | 0.2 | 692.7 | 1.97 | 0.07 | 537 | 14 | 8/10 | -- | gi\|13591997 | | (-)LFIDGK | |
| 316 | | 4.0e5 | 1601-1606 | 1 | 0.2 | 1062.9 | 1.94 | 0.24 | 307 | 7 | 9/14 | -- | gi\|13591997 | | (-)DMDLYSYR | |
| 471 | | 3.9e5 | 1982-1995 | 2 | -0.5 | 2281.0 | 1.94 | 0.20 | 399 | 1 | 14/42 | -- | gi\|13591997 | | (-)IVNDNPYGNGTAIFTTNGAIAR | |
| 418 | | 1.0e7 | 1792-1817 | 1 | 0.5 | 1225.9 | 1.94 | 0.17 | 84 | 3 | 11/18 | -- | gi\|13591997 | | (-)QGIQFYTQLK | |
| 238 | | 1.1e8 | 1276-1332 | 1 | 0.2 | 901.9 | 1.84 | 0.03 | 487 | 1 | 10/14 | -- | gi\|13591997 | | (-)LITLEQGK | |
| 306 | | 3.3e5 | 1589 | 2 | 0.2 | 1031.0 | 1.83 | 0.34 | 425 | 2 | 10/18 | -- | gi\|13591997 | | (-)EGASILLDGR | |
| 173 | | 5.3e7 | 0962-1010 | 1 | 0.2 | 901.8 | 1.76 | 0.22 | 151 | 2 | 7/14 | -- | gi\|13591997 | | (-)GDTNFYGK | |

# Flicka

Setup CreateDTA VuDTA RunSequest Status Summary Home

**Sample:** Berne, M. K6 1

**Send to:** PEPCUT PEPSTAT BLAST **NCBI:** SEQUENCE ABSTRACT

>**gi|13591997**|ref|NP_112319.1| methylmalonate semialdehyde dehydrogenase gene [Rattus norvegicus] gi|400269|sp|Q02253|MMSA_RAT Methylmalonate-semialdehyde dehydrogenase [acylating], mitochondrial precursor (MMSDH) gi|347034|pir||A44097 methylmalonate-semialdehyde dehydrogenase (acylating) (EC 1.2.1.27) - rat gi|205526|gb|AAA41638.1| methylmalonate semialdehyde dehydrogenase [MASS=57808] [MASS=57808]

```
MAAAVAAAAA VRSRILQVSS KVNSTWYPAS SFSSSSVPTV KLFIDGKFVE SKSDKWIDIH NPATNEVVGR VPQSTKAEME
AAVAACKRAF PAWADTSILS RQQVLLRYQQ LIKENLKEIA RLITLEQGKT LADAEGDVFR GLQVVEHACS VTSLMLGETM
PSITKDMDLY SYRLPLGVCA GIAPFNFPAM IPLWMFPMAM VCGNTFLMKP SERVPGATML LAKLLQDSGA PDGTLNIIHG
QHEAVNFICD HPDIKAISFV GSNQAGEYIF ERGSRNGKRV QANMGAKNHG VVMPDANKEN TLNQLVGAAF GAAGQRCMAL
STAVLVGEAK KWLPELVERA KNLRVNAGDQ PGADLGPLIT PQAKERVCNL IDSGAKEGAS ILLDGRKIKV KGYENGNFVG
PTIISNVKPS MTCYKEEIFG PVLVVLETET LDEAIKIVND NPYGNGTAIF TTNGAIARKY AHMVDVGQVG VNVPIPVPLP
MFSFTGSRSS FRGDTNFYGK QGIQFYTQLK TITSQWKEED ATLSSPAVVM PTMGR
```

**Mass (mono):** 57770.6 **Identifier:** gi|13591997 **Database:** C:/database/rat.fasta

**Protein Coverage:** 301/535 = **56.3%** by amino acid count, 32467.4/57770.6 = **56.2%** by mass

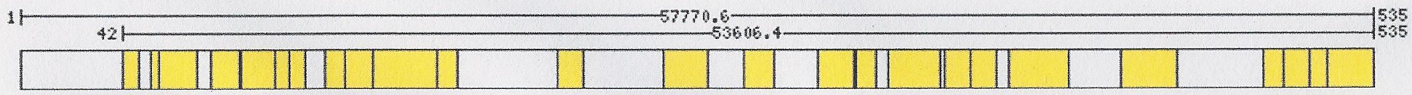


| Sort by: Sequence | Position |
|---|---|
| PEPSTAT AEMEAAVAACK | 77 - 87 |
| PEPSTAT AFPAWADTSILSR | 89 - 101 |
| PEPSTAT AISFVGSNQAGEYIFER | 256 - 272 |
| PEPSTAT CMALSTAVLVGEAK | 317 - 330 |
| PEPSTAT DMDLYSYR | 166 - 173 |
| PEPSTAT EEDATLSSPAVVMPTMGR | 518 - 535 |
| PEPSTAT EGASILLDGR | 377 - 386 |
| PEPSTAT GDTNFYGK | 493 - 500 |
| | 141 - 165 |

# Sequest Summary

File Select Sequest Protein Utilities Help

**Sample:** Berne, M. (K7) 1 mb
**DataFiles:** K7 (03/01/04-03/01/04)
**Database:** rat (01/05/2004)
**Directory:** mbernek7 View Info
**Diff Mods:** 16 M 57.02 C 0.000 X

**OutFiles:** 1380|925
**Enzyme:** Trypsin_Strict
**Mass:** ±1.5 (Mono)
**Max list:** 1000
**Intensity:** 8.4e9 MS2

DTA VCR

Consensus **Depth:** 3

A gi|25453414|ref|NP_037289.1| 228 42|Σ:2.8e9|33%|avg:6.6e7 {37,3,2,0,0,1}
arginosuccinate synthetase; arginosuccinate synthetase 1 [Rattus norvegicus] gi|114291|sp|P09034|ASSY_RAT Argininosuccinate synthase (Citrulline--aspartate ligase) gi|68637|pir||AJRTRS argininosuccinate synthase (EC 6.3.4.5) - rat gi|55767|emb|CAA30999.1| unnamed protei

| # | GBU | TIC | File | z | dM | MH+ | xC | dCn | Sp | RSp | Ions | Sf | Ref | () | Sequence | Prob |
|---|---|---|---|---|---|---|---|---|---|---|---|---|---|---|---|---|
| 921 | | 1.2e7 | 2715-2751 | 2 | -0.6 | 2315.7 | 5.65 | 0.61 | 1249 | 1 | 22/40 | -- | gi\|25453414 | | (-)DGTTHSTSLDLFM*YLNEVAGK | |
| 924 | | 5.3e7 | 2716-2741 | 3 | -0.7 | 2315.8 | 5.59 | 0.54 | 1806 | 1 | 32/80 | -- | gi\|25453414 | | (-)DGTTHSTSLDLFM*YLNEVAGK | |
| 761 | | 2.2e8 | 2329-2370 | 3 | -0.4 | 2693.6 | 5.43 | 0.52 | 2136 | 1 | 39/88 | -- | gi\|25453414 | | (-)SPWSM*DENLM*HISYEAGILENPK | |
| 658 | | 1.1e8 | 2049-2079 | 3 | 0.5 | 1601.3 | 5.43 | 0.50 | 1110 | 1 | 28/52 | -- | gi\|25453414 | | (-)APNTPDVLEIEFKK | |
| 883 | | 1.4e8 | 2613-2655 | 2 | 0.8 | 1782.2 | 5.38 | 0.57 | 1432 | 1 | 21/30 | -- | gi\|25453414 | | (-)EQGYDVIAYLANIGQK | |
| 851 | | 7.3e6 | 2501-2514 | 3 | -0.7 | 2677.9 | 4.91 | 0.14 | 1133 | 1 | 29/88 | -- | gi\|25453414 | | (-)SPWSM*DENLMHISYEAGILENPK | |
| 868 | | 2.3e7 | 2577-2614 | 2 | -0.7 | 2374.8 | 4.72 | 0.51 | 1079 | 1 | 18/36 | -- | gi\|25453414 | | (-)FAELVYTGFWHSPEC#EFVR | |
| 762 | | 1.3e7 | 2338-2352 | 2 | -1.1 | 2694.3 | 4.59 | 0.47 | 645 | 1 | 18/44 | -- | gi\|25453414 | | (-)SPWSM*DENLM*HISYEAGILENPK | |
| 887 | | 2.8e6 | 2622-2650 | 3 | 0.5 | 1782.5 | 4.56 | 0.34 | 1442 | 1 | 29/60 | -- | gi\|25453414 | | (-)EQGYDVIAYLANIGQK | |
| 938 | | 3.3e5 | 2779-2791 | 2 | 0.8 | 2299.8 | 4.24 | 0.54 | 1152 | 1 | 19/40 | -- | gi\|25453414 | | (-)DGTTHSTSLDLFMYLNEVAGK | |
| 655 | | 6.1e7 | 2044-2082 | 2 | 1.4 | 1600.5 | 4.15 | 0.47 | 630 | 1 | 17/26 | -- | gi\|25453414 | | (-)APNTPDVLEIEFKK | |
| 733 | | 5.9e6 | 2259-2260 | 2 | 0.7 | 1472.9 | 3.88 | 0.53 | 848 | 1 | 17/24 | -- | gi\|25453414 | | (-)APNTPDVLEIEFK | |
| 719 | | 5.9e8 | 2207-2254 | 2 | 0.3 | 1473.3 | 3.76 | 0.40 | 649 | 1 | 17/24 | -- | gi\|25453414 | | (-)APNTPDVLEIEFK | |
| 1007 | | 6.5e6 | 3030-3034 | 3 | -1.5 | 2851.9 | 3.28 | 0.23 | 381 | 1 | 26/96 | -- | gi\|25453414 | | (-)GIYETPAGTILYHAHLDIEAFTM*DR | |
| 534 | | 1.1e8 | 1811-1835 | 2 | -1.2 | 995.4 | 3.23 | 0.20 | 867 | 1 | 15/16 | -- | gi\|25453414 | | (-)YLLGTSLAR | |
| 439 | | 7.0e7 | 1420-1456 | 2 | 0.4 | 1064.8 | 3.04 | 0.39 | 725 | 1 | 14/16 | -- | gi\|25453414 | | (-)KVFIEDVSK | |
| 454 | | 3.5e7 | 1517-1537 | 2 | 0.1 | 937.0 | 2.82 | 0.26 | 443 | 4 | 12/14 | -- | gi\|25453414 | | (-)VFIEDVSK | |
| 475 | | 8.7e7 | 1588-1621 | 2 | 0.0 | 906.1 | 2.66 | 0.43 | 718 | 1 | 12/14 | -- | gi\|25453414 | | (-)GQVYILGR | |
| 366 | | 5.9e6 | 1162 | 2 | -0.4 | 1213.0 | 2.55 | 0.37 | 564 | 1 | 14/18 | -- | gi\|25453414 | +1 | (-)GRNDLM*EYAK | |
| 274 | | 2.6e7 | 0845-0868 | 2 | -1.1 | 973.3 | 2.44 | 0.10 | 531 | 1 | 11/14 | -- | gi\|25453414 | | (-)KQVEIAQR | |
| 374 | | 8.7e7 | 1183-1219 | 2 | -0.8 | 1000.2 | 2.37 | 0.23 | 901 | 1 | 13/14 | -- | gi\|25453414 | +1 | (-)NDLM*EYAK | |
| 441 | | 1.6e6 | 1429-1447 | 1 | 0.9 | 1064.4 | 2.29 | 0.29 | 596 | 1 | 11/16 | -- | gi\|25453414 | | (-)KVFIEDVSK | |
| 389 | | 7.3e7 | 1225-1255 | 2 | -0.5 | 972.9 | 2.16 | 0.32 | 698 | 1 | 12/12 | -- | gi\|25453414 | +1 | (-)M*PEFYNR | |
| 456 | | 7.3e7 | 1525-1543 | 2 | 0.1 | 806.9 | 2.15 | 0.08 | 410 | 1 | 11/12 | -- | gi\|25453414 | | (-)VQVSVFK | |
| 452 | | 5.7e7 | 1506-1540 | 1 | 0.6 | 936.5 | 2.11 | 0.21 | 645 | 2 | 10/14 | -- | gi\|25453414 | | (-)VFIEDVSK | |
| 453 | | 1.5e8 | 1512-1555 | 1 | 0.1 | 806.8 | 2.08 | 0.15 | 792 | 1 | 10/12 | -- | gi\|25453414 | | (-)VQVSVFK | |
| 395 | | 1.5e7 | 1267-1273 | 2 | -0.8 | 973.2 | 2.03 | 0.15 | 474 | 1 | 10/12 | -- | gi\|25453414 | +1 | (-)M*PEFYNR | |
| 306 | | 2.8e7 | 1003-1031 | 2 | 0.6 | 895.3 | 2.00 | 0.17 | 580 | 1 | 11/12 | -- | gi\|25453414 | | (-)EDFEEAR | |
| 492 | | 1.1e6 | 1656-1669 | 1 | 0.9 | 1186.5 | 1.95 | 0.39 | 93 | 1 | 10/20 | -- | gi\|25453414 | | (-)QHGIPIPVTPK | |
| 212 | | 1.5e7 | 0520-0543 | 2 | 0.4 | 845.6 | 1.92 | 0.02 | 359 | 3 | 9/10 | -- | gi\|25453414 | | (-)LKEYHR | |
| 491 | | 1.7e8 | 1650-1681 | 3 | -0.1 | 1187.5 | 1.87 | 0.02 | 471 | 1 | 18/40 | -- | gi\|25453414 | | (-)QHGIPIPVTPK | |
| 473 | | 1.7e8 | 1581-1627 | 1 | 0.2 | 905.9 | 1.78 | 0.08 | 208 | 44 | 9/14 | -- | gi\|25453414 | | (-)GQVYILGR | |
| 488 | | 8.9e7 | 1649-1684 | 2 | 0.2 | 1187.2 | 1.77 | 0.07 | 293 | 1 | 14/20 | -- | gi\|25453414 | | (-)QHGIPIPVTPK | |

| | | | | | | | | | | | | | | |
|---|---|---|---|---|---|---|---|---|---|---|---|---|---|---|
| 296 | 3.8e7 | 0941-0970 | 2 | 0.4 | 843.6 | 1.61 | ---- | 445 | 1 | 12/12 | -- | gi\|25453414 | | (-)QVEIAQR |
| 359 | 2.2e7 | 1139-1210 | 1 | -0.1 | 999.6 | 1.58 | 0.12 | 104 | 1 | 9/14 | -- | gi\|25453414 | +1 | (-)NDLM*EYAK |
| 298 | 2.3e7 | 0946-0972 | 1 | 0.1 | 843.9 | 1.57 | 0.02 | 83 | 3 | 9/12 | -- | gi\|25453414 | | (-)QVEIAQR |
| 458 | 2.5e7 | 1541-1573 | 1 | 0.5 | 741.4 | 1.32 | ---- | 138 | 72 | 6/10 | -- | gi\|25453414 | +1 | (-)VIAPWR |
| 532 | 6.5e7 | 1807-1843 | 1 | 0.2 | 994.0 | 1.26 | 0.11 | 68 | 13 | 9/16 | -- | gi\|25453414 | | (-)YLLGTSLAR |
| 397 | 3.5e6 | 1272-1290 | 1 | 0.8 | 1088.4 | 1.03 | ---- | 234 | 1 | 11/18 | -- | gi\|25453414 | | (-)NQAPPGLYTK |
| 393 | 1.5e7 | 1241-1253 | 1 | 0.0 | 972.4 | 1.09 | 0.15 | 46 | 4 | 7/12 | -- | gi\|25453414 | +1 | (-)M*PEFYNR |
| 311 | 5.9e7 | 1020-1059 | 1 | 0.0 | 615.8 | 0.93 | ---- | 62 | 1 | 8/10 | -- | gi\|25453414 | | (-)QGLGLK |
| 206 | 2.5e5 | 0502 | 1 | 0.6 | 627.2 | 0.77 | ---- | 102 | 19 | 4/10 | -- | gi\|25453414 | | (-)KGVPVK |

⊟ B gi|8392833|ref|NP_058682.1| 132 18|Σ:2.6e8|3%|avg:1.5e7 {16,0,2,0,0,2}
acetyl-coenzyme A dehydrogenase, medium chain; Acyl-Coenzyme A dehydrogenase, C-4 to C-12 straight-chain [Rattus norvegicus] gi|113018|sp|P08503|ACDM_RAT Acyl-CoA dehydrogenase, medium-chain specific, mitochondrial precursor (MCAD) gi|66079|pir||DERTCM acyl-CoA dehydroge

| | | | | | | | | | | | | | | |
|---|---|---|---|---|---|---|---|---|---|---|---|---|---|---|
| 767 | 3.4e7 | 2351-2380 | 2 | 0.6 | 1873.6 | 5.31 | 0.62 | 1981 | 1 | 24/34 | -- | gi\|8392833 | | (-)AFTGFIVEADTPGIHIGK |
| 459 | 6.4e7 | 1547-1577 | 2 | 0.2 | 1468.5 | 4.55 | 0.48 | 1202 | 1 | 22/28 | -- | gi\|8392833 | | (-)TRPTVAAGAVGLAQR |
| 736 | 5.4e7 | 2265-2307 | 2 | 0.5 | 1838.5 | 4.32 | 0.52 | 671 | 1 | 23/30 | -- | gi\|8392833 | | (-)QEPGLGFSFELTEQQK |
| 738 | 8.8e5 | 2266-2280 | 2 | -0.9 | 2049.0 | 4.13 | 0.55 | 716 | 1 | 19/34 | -- | gi\|8392833 | | (-)LLVEHQGVSFLLAEM*AM*K |
| 466 | 2.3e7 | 1553-1570 | 3 | 0.7 | 1468.0 | 3.94 | 0.23 | 1015 | 1 | 26/56 | -- | gi\|8392833 | | (-)TRPTVAAGAVGLAQR |
| 346 | 7.5e6 | 1115-1146 | 2 | 0.2 | 1251.2 | 3.94 | 0.32 | 1509 | 1 | 16/20 | -- | gi\|8392833 | | (-)KGDEYVINGQK |
| 530 | 2.9e6 | 1806 | 2 | 0.7 | 1583.1 | 3.69 | 0.39 | 1512 | 1 | 19/24 | -- | gi\|8392833 | | (-)IYQIYEGTAQIQR |
| 741 | 1.9e6 | 2267 | 3 | -1.1 | 2049.2 | 3.01 | 0.25 | 568 | 1 | 21/68 | -- | gi\|8392833 | | (-)LLVEHQGVSFLLAEM*AM*K |
| 765 | 1.0e6 | 2347 | 2 | 0.4 | 1170.0 | 2.75 | 0.51 | 1120 | 1 | 14/16 | -- | gi\|8392833 | | (-)ANWYFVLTR |
| 688 | 2.8e7 | 2130-2154 | 2 | -0.1 | 1151.5 | 2.44 | 0.25 | 484 | 1 | 13/18 | -- | gi\|8392833 | | (-)SGEYPFPLIK |
| 407 | 4.5e6 | 1330-1356 | 2 | -0.3 | 991.3 | 2.43 | 0.33 | 963 | 2 | 14/16 | -- | gi\|8392833 | | (-)AAWEVDSGR |
| 536 | 3.6e6 | 1839 | 2 | 0.4 | 1234.0 | 2.42 | 0.33 | 461 | 1 | 15/22 | -- | gi\|8392833 | | (-)ENVLIGEGAGFK |
| 770 | 9.4e5 | 2353-2376 | 3 | 0.4 | 1873.8 | ~~2.03~~ | ~~0.10~~ | ~~246~~ | ~~13~~ | 17/68 | -- | gi\|8392833 | | (-)AFTGFIVEADTPGIHIGK |
| 813 | 6.0e5 | 2448 | 2 | -2.0 | 1876.1 | 1.86 | 0.14 | 280 | 1 | 11/34 | -- | gi\|8392833 | | (-)AFTGFIVEADTPGIHIGK |
| 214 | 1.6e7 | 0525-0555 | 1 | 0.1 | 747.7 | 1.70 | 0.06 | 377 | 2 | 8/12 | -- | gi\|8392833 | | (-)ALDEATK |
| 494 | 1.3e7 | 1668-1687 | 1 | 0.5 | 1014.7 | 1.67 | 0.20 | 369 | 1 | 11/16 | -- | gi\|8392833 | | (-)NTYFASIAK |
| 391 | 5.9e6 | 1231-1237 | 1 | 0.6 | 864.4 | 1.37 | ---- | 144 | 21 | 8/12 | -- | gi\|8392833 | | (-)EFQTIAR |
| 1258 | 1.8e5 | 3792 | 2 | -1.3 | 1929.5 | 1.14 | ---- | 137 | 9 | 10/32 | -- | gi\|8392833 | | (-)ALDEATKYALDRKTFGK |

⊟ C gi|1730519|sp|P50310|PGK_CRIGR 134 18|Σ:1.1e8|1%|avg:6.3e6 {14,4,0,0,0,0}
Phosphoglycerate kinase gi|2117894|pir||I48074 phosphoglycerate kinase (EC 2.7.2.3) - Chinese hamster gi|987048|emb|CAA86028.1| phosphoglycerate kinase [Cricetulus griseus] [MASS=44563] [MASS=44563]

| | | | | | | | | | | | | | | |
|---|---|---|---|---|---|---|---|---|---|---|---|---|---|---|
| 570 | 1.0e7 | 1913-1935 | 2 | 0.8 | 1741.2 | 4.84 | 0.62 | 1620 | 1 | 25/34 | -- | gi\|1730519 | +29 | (-)VSHVSTGGGASLELLEGK |
| 782 | 3.3e6 | 2401-2424 | 2 | 0.9 | 2023.3 | 4.75 | 0.36 | 1102 | 1 | 21/34 | -- | gi\|1730519 | +10 | (-)ITLPVDFVTADKFDENAK |
| 553 | 2.3e7 | 1879-1894 | 3 | 0.2 | 1635.6 | 4.49 | 0.46 | 1231 | 1 | 29/56 | -- | gi\|1730519 | +35 | (-)LGDVYVNDAFGTAHR |
| 847 | 8.3e6 | 2494-2512 | 2 | 0.2 | 1769.9 | 4.42 | ---- | 1277 | 1 | 21/32 | -- | gi\|1730519 | +20 | (-)ALESPERPFLAILGGAK |
| 842 | 1.0e7 | 2487-2511 | 3 | 0.7 | 1769.4 | 4.34 | 0.24 | 1689 | 1 | 37/64 | -- | gi\|1730519 | +20 | (-)ALESPERPFLAILGGAK |
| 550 | 1.7e7 | 1878-1895 | 2 | 0.6 | 1635.2 | 4.04 | 0.36 | 1497 | 1 | 20/28 | -- | gi\|1730519 | +35 | (-)LGDVYVNDAFGTAHR |
| 575 | 3.7e6 | 1925-1932 | 3 | 0.1 | 1741.9 | 3.66 | 0.48 | 1466 | 1 | 30/68 | -- | gi\|1730519 | +29 | (-)VSHVSTGGGASLELLEGK |
| 538 | 9.9e5 | 1840-1861 | 2 | -1.0 | 2000.0 | 3.45 | 0.33 | 526 | 1 | 16/34 | -- | gi\|1730519 | +6 | (-)VLNNM*EIGTSLYDEEGAK |
| 425 | 6.9e6 | 1375-1385 | 2 | 0.1 | 1383.6 | 3.30 | ---- | 1142 | 1 | 17/24 | -- | gi\|1730519 | +13 | (-)AHSSM*VGVNLPQK |
| 218 | 1.6e6 | 0548 | 2 | 0.1 | 1102.1 | 2.96 | 0.12 | 881 | 1 | 14/16 | -- | gi\|1730519 | +13 | (-)NNQITNNQR |
| 606 | 8.5e6 | 1972-1985 | 2 | -0.8 | 1221.2 | 2.79 | 0.36 | 492 | 1 | 15/20 | -- | gi\|1730519 | +9 | (-)YSLEPVAAELK |
| 509 | 6.6e5 | 1740-1747 | 2 | -0.5 | 2067.5 | 2.28 | 0.06 | 193 | 1 | 14/36 | -- | gi\|1730519 | +8 | (-)SVVLM*SHLGRPDGVPM*PDK |
| 554 | 1.0e7 | 1885-1908 | 1 | 0.5 | 734.4 | 1.78 | ---- | 494 | 3 | 8/10 | -- | gi\|1730519 | +18 | (-)DVLFLK |
| 785 | 7.9e6 | 2411-2417 | 3 | -0.9 | 2025.1 | 1.72 | 0.11 | 360 | 1 | 20/68 | -- | gi\|1730519 | +10 | (-)ITLPVDFVTADKFDENAK |

# Flicka



**Sample:** Berne, M. K7 1

**Send to:** PEPCUT PEPSTAT BLAST **NCBI:** SEQUENCE ABSTRACT

>**gi|25453414**|ref|NP_037289.1| arginosuccinate synthetase; arginosuccinate synthetase 1 [Rattus norvegicus] gi|114291|sp|P09034|ASSY_RAT Argininosuccinate synthase (Citrulline--aspartate ligase) gi|68637|pir||AJRTRS argininosuccinate synthase (EC 6.3.4.5) - rat gi|55767|emb|CAA30999.1| unnamed protein product [Rattus norvegicus] gi|203016|gb|AAA40771.1| argininosuccinate synthetase [Rattus norvegicus] [MASS=46496] [MASS=46496]

```
MSSKGSVVLA YSGGLDTSCI LVWLKEQGYD VIAYLANIGQ KEDFEEARKK ALKLGAKKVF IEDVSKEFVE EFIWPAVQSS
ALYEDRYLLG TSLARPCIAR KQVEIAQREG AKYVSHGATG KGNDQVRFEL TCYSLAPQIK VIAPWRMPEF YNRFKGRNDL
MEYAKQHGIP IPVTPKSPWS MDENLMHISY EAGILENPKN QAPPGLYTKT QDPAKAPNTP DVLEIEFKKG VPVKVTNVKD
GTTHSTSLDL FMYLNEVAGK HGVGRIDIVE NRFIGMKSRG IYETPAGTIL YHAHLDIEAF TMDREVRKIK QGLGLKFAEL
VYTGFWHSPE CEFVRHCIDK SQERVEGKVQ VSVFKGQVYI LGRESPLSLY NEELVSMNVQ GDYEPIDATG FININSLRLK
EYHRLQSKVT AK
```

**Mass (mono):** 46466.9 **Identifier:** gi|25453414 **Database:** C:/database/rat.fasta
**Protein Coverage:** 227/412 = **55.1%** by amino acid count, 25899.2/46466.9 = **55.7%** by mass

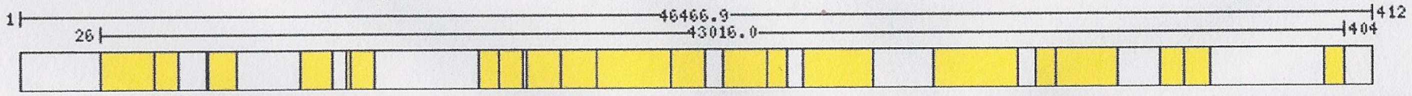


| Sort by: Sequence | Position |
|---|---|
| PEPSTAT APNTPDVLEIEFK | 216 - 228 |
| PEPSTAT APNTPDVLEIEFKK | 216 - 229 |
| PEPSTAT DGTTHSTSLDLFMYLNEVAGK | 240 - 260 |
| PEPSTAT EDFEEAR | 42 - 48 |
| PEPSTAT EQGYDVIAYLANIGQK | 26 - 41 |
| PEPSTAT FAELVYTGFWHSPECEFVR | 317 - 335 |
| PEPSTAT GIYETPAGTILYHAHLDIEAFTMDR | 280 - 304 |
| PEPSTAT GQVYILGR | 356 - 363 |
| PEPSTAT GRNDLMEYAK | 156 - 165 |

# Flicka



**Sample:** Berne, M. K7 1

**Send to:** PEPCUT PEPSTAT BLAST **NCBI:** SEQUENCE ABSTRACT

>**gi|1730519**|sp|P50310|PGK_CRIGR Phosphoglycerate kinase gi|2117894|pir||I48074 phosphoglycerate kinase (EC 2.7.2.3) - Chinese hamster gi|987048|emb|CAA86028.1| phosphoglycerate kinase [Cricetulus griseus] [MASS=44563] [MASS=44563]

MSLSNKLTLD KLDVKGKRVV MRVDFNVPMK **NNQITNNQR**I KAAVPSIKFC LDNGAK**SVVL MSHLGRPDGV PMPDKYSLEP**
**VAAELK**SLLG K**DVLFLK**DCV GPEVENACAN PAAGTVILLE NLRFHVEEEG KGKDASGNKI KAEPAKIDAF RASLSK**LGDV**
**YVNDAFGTAH RAHSSMVGVN LPQK**AGGFLM KKELNYFAK**A LESPERPFLA ILGGAK**VADK IQLINNMLDK VNEMIIGGGM
AFTFLK**VLNN MEIGTSLYDE EGAK**IVKDLM AKAEKNGVK**I TLPVDFVTAD KFDENAK**TGQ ATVASGIPAG WMGLDCGTES
SKK**YAEAVAR** AK**QIVWNGPV GVFEWEAFAR** GTKSLMDEVV KATSRGCITI IGGGDTATCC AK**WNTEDKVS HVSTGGGASL**
**ELLEGKVLPG VDALSNV**

**Mass (mono):** 44534.0 **Identifier:** gi|1730519 **Database:** C:/database/rat.fasta
**Protein Coverage:** 186/417 = **44.6%** by amino acid count, 20121.3/44534.0 = **45.2%** by mass

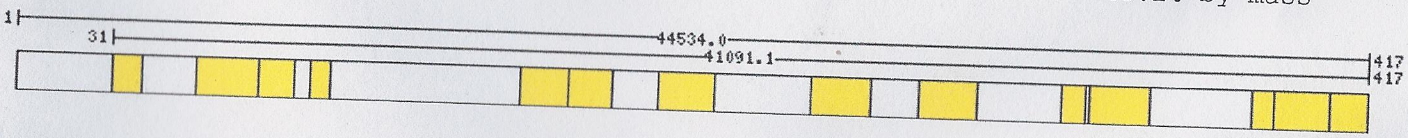


**Sort by:** Sequence ☐ Position ☐

| | Sequence | Position |
|---|---|---|
| PEPSTAT | AHSSMVGVNLPQK | 172 - 184 |
| PEPSTAT | ALESPERPFLAILGGAK | 200 - 216 |
| PEPSTAT | DVLFLK | 92 - 97 |
| PEPSTAT | ITLPVDFVTADKFDENAK | 280 - 297 |
| PEPSTAT | LGDVYVNDAFGTAHR | 157 - 171 |
| PEPSTAT | NNQITNNQR | 31 - 39 |
| PEPSTAT | QIVWNGPVGVFEWEAFAR | 333 - 350 |
| PEPSTAT | SVVLMSHLGRPDGVPMPDK | 57 - 75 |
| PEPSTAT | VLNNMEIGTSLYDEEGAK | 247 - 264 |
| PEPSTAT | VLPGVDALSNV | 407 - 417 |

# Flicka



**Sample:** Berne, M. K7 1

**Send to:** PEPCUT PEPSTAT BLAST **NCBI:** SEQUENCE ABSTRACT

>**gi|8392833**|ref|NP_058682.1| acetyl-coenzyme A dehydrogenase, medium chain; Acyl-Coenzyme A dehydrogenase, C-4 to C-12 straight-chain [Rattus norvegicus] gi|113018|sp|P08503|ACDM_RAT Acyl-CoA dehydrogenase, medium-chain specific, mitochondrial precursor (MCAD) gi|66079|pir||DERTCM acyl-CoA dehydrogenase (EC 1.3.99.3) precursor, medium-chain-specific, mitochondrial - rat gi|202689|gb|AAA40670.1| acyl-CoA dehydrogenase medium subunit (EC 1.3.99.3) [MASS=46555] [MASS=46555]

```
MAAALRRGYK VLRSVSHFEC RAQHTKPSLK QEPGLGFSFE LTEQQKEFQT IARKFAREEI IPVAPDYDKS GEYPFPLIKR
AWELGLINTH IPESCGGLGL GTFDACLITE ELAYGCTGVQ TAIEANSLGQ MPVIIAGNDQ QKKKYLGRMT EQPMMCAYCV
TEPSAGSDVA GIKTKAEKKG DEYVINGQKM WITNGGKANW YFVLTRSNPD PKVPASKAFT GFIVEADTPG IHIGKKELNM
GQRCSDTRGI TFEDVRVPKE NVLIGEGAGF KIAMGAFDRT RPTVAAGAVG LAQRALDEAT KYALDRKTFG KLLVEHQGVS
FLLAEMAMKV ELARLSYQRA AWEVDSGRRN TYFASIAKAF AGDIANQLAT DAVQIFGGYG FNTEYPVEKL MRDAKIYQIY
EGTAQIQRLI IAREHIEKYK N
```

**Mass (mono):** 46525.8 **Identifier:** gi|8392833 **Database:** C:/database/rat.fasta

**Protein Coverage:** 164/421 = **39.0%** by amino acid count, 18149.4/46525.8 = **39.0%** by mass

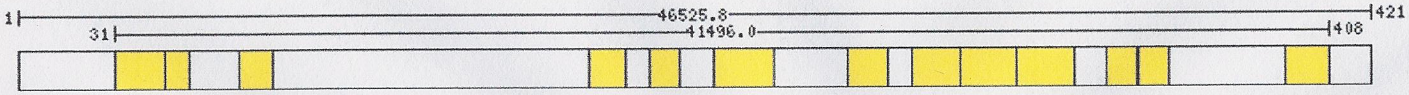


**Sort by:** **Sequence** **Position**

| | Sequence | Position |
|---|---|---|
| PEPSTAT | AAWEVDSGR | 340 - 348 |
| PEPSTAT | AFTGFIVEADTPGIHIGK | 218 - 235 |
| PEPSTAT | ALDEATK | 295 - 301 |
| PEPSTAT | ALDEATKYALDRKTFGK | 295 - 311 |
| PEPSTAT | ANWYFVLTR | 198 - 206 |
| PEPSTAT | EFQTIAR | 47 - 53 |
| PEPSTAT | ENVLIGEGAGFK | 260 - 271 |
| PEPSTAT | IYQIYEGTAQIQR | 396 - 408 |
| | | 179 - 189 |

# Sequest Summary

File Select Sequest Protein Utilities Help

**Sample:** Berne, M. (K11) 1 mb
**DataFiles:** K11 (03/02/04-03/02/04)
**Database:** rat (01/05/2004)
**Directory:** mbernek11 View Info
**Diff Mods:** 16 M 57.02 C 0.000 X

**OutFiles:** 1142|754
**Enzyme:** Trypsin_Strict
**Mass:** ±1.5 (Mono)
**Max list:** 1000
**Intensity:** 1.4e10 MS2 DTA VCR

Consensus Depth: 3

A gi|2119726|pir||I56581 302 61|Σ:4.9e9|35%|avg:8.0e7 {54,6,1,0,2,4}
dnaK-type molecular chaperone grp75 precursor - rat gi|1000439|gb|AAB34982.1| grp75 [Rattus sp.] [MASS=73745] [MASS=73745]

| # | GBU | TIC | File | z | dM | MH+ | xC | dCn | Sp | RSp | Ions | Sf | Ref | () | Sequence | Prob |
|---|---|---|---|---|---|---|---|---|---|---|---|---|---|---|---|---|
| 462 | | 2.5e7 | 1838-1849 | 2 | -0.1 | 1810.1 | 5.71 | 0.56 | 2171 | 1 | 24/32 | -- | gi\|2119726 | +1 | (-) SQVFSTAADGQTQVEIK | |
| 566 | | 4.0e8 | 2188-2229 | 2 | 0.3 | 1646.6 | 5.52 | ---- | 1241 | 1 | 24/30 | -- | gi\|2119726 | +3 | (-) VINEPTAAALAYGLDK | |
| 476 | | 2.7e7 | 1887-1918 | 2 | 0.6 | 1809.4 | 5.44 | 0.54 | 2342 | 1 | 24/32 | -- | gi\|2119726 | +1 | (-) SQVFSTAADGQTQVEIK | |
| 491 | | 1.0e7 | 1930-1958 | 2 | 0.6 | 1809.4 | 5.25 | 0.52 | 1533 | 1 | 22/32 | -- | gi\|2119726 | +1 | (-) SQVFSTAADGQTQVEIK | |
| 514 | | 3.8e8 | 2013-2053 | 2 | -0.8 | 1873.7 | 4.57 | 0.51 | 1472 | 1 | 21/32 | -- | gi\|2119726 | +1 | (-) VEAVNM*AEGIIHDTETK | |
| 649 | | 1.9e7 | 2440-2464 | 2 | -0.3 | 2642.6 | 4.45 | 0.62 | 606 | 1 | 20/46 | -- | gi\|2119726 | +2 | (-) C#ELSSSVQTDINLPYLTM*DASGPK | |
| 509 | | 1.7e8 | 1992-2024 | 2 | 0.1 | 1243.3 | 4.33 | 0.35 | 1174 | 1 | 20/22 | -- | gi\|2119726 | +2 | (-) DAGQISGLNVLR | |
| 385 | | 7.3e7 | 1624-1654 | 2 | 0.6 | 1474.1 | 4.20 | 0.51 | 1460 | 1 | 22/26 | -- | gi\|2119726 | +1 | (-) EQQIVIQSSGGLSK | |
| 667 | | 1.4e8 | 2510-2542 | 2 | -0.1 | 1569.9 | 4.13 | 0.48 | 1130 | 1 | 20/26 | -- | gi\|2119726 | +2 | (-) LYSPSQIGAFVLM*K | |
| 362 | | 2.4e7 | 1570-1582 | 2 | -0.6 | 1870.4 | 4.05 | 0.53 | 1147 | 1 | 21/28 | -- | gi\|2119726 | +1 | (-) M*EEFKDQLPADEC#NK | |
| 672 | | 3.4e8 | 2534-2571 | 2 | 0.2 | 1334.4 | 4.04 | 0.42 | 1541 | 1 | 19/22 | -- | gi\|2119726 | +5 | (-) AQFEGIVTDLIK | |
| 739 | | 6.1e5 | 2773-2776 | 2 | 0.9 | 2309.7 | 4.02 | 0.56 | 957 | 1 | 21/42 | -- | gi\|2119726 | +1 | (-) GVPQIEVTFDIDANGIVHVSAK | |
| 500 | | 2.9e8 | 1956-1994 | 2 | 0.6 | 1290.9 | 3.99 | 0.45 | 1426 | 1 | 17/20 | -- | gi\|2119726 | +1 | (-) VQQTVQDLFGR | |
| 533 | | 3.4e8 | 2077-2112 | 2 | -0.4 | 1463.2 | 3.89 | 0.32 | 786 | 1 | 18/26 | -- | gi\|2119726 | +2 | (-) SDIGEVILVGGM*TR | |
| 322 | | 8.4e6 | 1383-1389 | 2 | 0.0 | 1334.4 | 3.88 | 0.47 | 975 | 1 | 16/22 | -- | gi\|2119726 | +3 | (-) ETAENYLGHTAK | |
| 517 | | 1.2e8 | 2025-2055 | 3 | -1.0 | 1874.0 | 3.83 | 0.43 | 688 | 1 | 29/64 | -- | gi\|2119726 | +1 | (-) VEAVNM*AEGIIHDTETK | |
| 713 | | 7.1e7 | 2637-2656 | 2 | 0.5 | 1593.5 | 3.81 | 0.55 | 766 | 1 | 21/28 | -- | gi\|2119726 | +1 | (-) LLGQFTLIGIPPAPR | |
| 310 | | 2.6e7 | 1310-1330 | 2 | -0.8 | 1609.6 | 3.81 | 0.49 | 1291 | 1 | 20/26 | -- | gi\|2119726 | +3 | (-) M*KETAENYLGHTAK | |
| 358 | | 3.6e7 | 1528-1533 | 2 | 0.3 | 1569.4 | 3.80 | 0.45 | 644 | 1 | 17/26 | -- | gi\|2119726 | +2 | (-) QAVTNPNNTFYATK | |
| 528 | | 1.2e7 | 2062-2073 | 2 | -0.7 | 1244.2 | 3.70 | 0.26 | 1384 | 1 | 20/22 | -- | gi\|2119726 | +2 | (-) DAGQISGLNVLR | |
| 316 | | 3.8e7 | 1353-1370 | 2 | 0.0 | 1232.4 | 3.69 | 0.43 | 806 | 1 | 17/22 | -- | gi\|2119726 | +2 | (-) QAASSLQQASLK | |
| 303 | | 1.0e7 | 1292-1298 | 2 | 0.1 | 1342.3 | 3.65 | 0.42 | 1129 | 1 | 18/24 | -- | gi\|2119726 | +2 | (-) ASNGDAWVEAHGK | |
| 466 | | 4.0e8 | 1846-1885 | 2 | 0.5 | 1477.1 | 3.55 | 0.61 | 695 | 1 | 17/26 | -- | gi\|2119726 | +2 | (-) TTPSVVAFTPDGER | |
| 728 | | 3.3e7 | 2742-2775 | 2 | 0.1 | 1593.9 | 3.49 | 0.36 | 734 | 1 | 20/28 | -- | gi\|2119726 | +1 | (-) LLGQFTLIGIPPAPR | |
| 718 | | 1.8e8 | 2692-2737 | 2 | 0.6 | 1593.4 | 3.46 | 0.45 | 577 | 1 | 19/28 | -- | gi\|2119726 | +1 | (-) LLGQFTLIGIPPAPR | |
| 204 | | 1.6e8 | 0954-0993 | 2 | -0.2 | 959.2 | 3.28 | 0.34 | 661 | 1 | 14/16 | -- | gi\|2119726 | +2 | (-) VLENAEGAR | |
| 557 | | 4.3e8 | 2155-2192 | 2 | 0.3 | 1695.6 | 3.23 | 0.09 | 577 | 1 | 20/28 | -- | gi\|2119726 | +2 | (-) NAVITVPAYFNDSQR | |
| 695 | | 4.9e6 | 2606-2612 | 2 | -0.6 | 1335.1 | 3.17 | 0.34 | 1325 | 1 | 17/22 | -- | gi\|2119726 | +5 | (-) AQFEGIVTDLIK | |
| 469 | | 1.1e7 | 1862-1876 | 1 | 0.6 | 1477.0 | 3.08 | 0.39 | 337 | 1 | 16/26 | -- | gi\|2119726 | +2 | (-) TTPSVVAFTPDGER | |
| 432 | | 1.4e6 | 1744 | 2 | 0.4 | 1474.2 | 3.08 | 0.39 | 661 | 1 | 16/26 | -- | gi\|2119726 | +1 | (-) EQQIVIQSSGGLSK | |
| 524 | | 8.8e6 | 2060-2068 | 2 | -1.5 | 1293.0 | 3.02 | 0.30 | 1422 | 1 | 17/20 | -- | gi\|2119726 | +1 | (-) VQQTVQDLFGR | |
| 391 | | 1.5e6 | 1636 | 1 | 1.0 | 1473.6 | 2.98 | 0.47 | 434 | 1 | 15/26 | -- | gi\|2119726 | +1 | (-) EQQIVIQSSGGLSK | |
| 214 | | 1.6e8 | 0990-1022 | 2 | 0.0 | 1150.2 | 2.92 | 0.23 | 502 | 1 | 13/16 | -- | gi\|2119726 | +2 | (-) RYDDPEVQK | |
| 569 | | 6.2e6 | 2206-2217 | 2 | 0.1 | 1695.8 | 2.85 | ---- | 387 | 1 | 14/28 | -- | gi\|2119726 | +2 | (-) NAVITVPAYFNDSQR | |

# Flicka

Setup CreateDTA VuDTA RunSequest Status Summary Home

**Sample:** Berne, M. K11 1

**Send to:** PEPCUT PEPSTAT BLAST **NCBI:** SEQUENCE ABSTRACT

>gi|2119726|pir||I56581 dnaK-type molecular chaperone grp75 precursor - rat gi|1000439|gb|AAB34982.1| grp75 [Rattus sp.] [MASS=73745] [MASS=73745]

MISASRAAAA RLVGTTASRS PAAARHQDGW NGLSHEAFRF VSRRDYASEA IKGAVVGIDL GTTNSCVAVM EGKQAKVLEN
AEGARTTPSV VAFTPDGERL VGMPAKRQAV TNPNNTFYAT KRLIGRRYDD PEVQKDTKNV PFKIVRASNG DAWVEAHGKL
YSPSQIGAFV LMKMKETAEN YLGHTAKNAV ITVPAYFNDS QRQATKDAGQ ISGLNVLRVI NEPTAAALAY GLDKSEDKVI
AVYDLGGGTF DISILEIQKG VFEVKSTNGD TFLGGEDFDQ ALLRHIVKEF KRETGVDLTK DNMALQRVRE AAEKAKCELS
SSVQTDINLP YLTMDASGPK HLNMKLTRAQ FEGIVTDLIK RTIAPCQKAM QDAEVSKSDI GEVILVGGMT RMPKVQQTVQ
DLFGRAPSKA VNPDEAVAIG AAIQGGVLAG DVTDVLLLDV TPLSLGIETL GGVFTKLINR NTTIPTKKSQ VFSTAADGQT
QVEIKVCQGE REMAGDNKLL GQFTLIGIPP APRGVPQIEV TFDIDANGIV HVSAKDKGTG REQQIVIQSS GGLSKDDIEN
MVKNAEKYAE EDRRKKERVE AVNMAEGIIH DTETKMEEFK DQLPADECNK LKEEISKMRE LLARKDSETG ENIRQAASSL
QQASLKLFEM AYKKMASERE GSGSSSTGEQ KEDQKEEKQ

**Mass (mono):** 73698.8 **Identifier:** gi|2119726 **Database:** C:/database/rat.fasta

**Protein Coverage:** 361/679 = **53.2%** by amino acid count, 39119.9/73698.8 = **53.1%** by mass

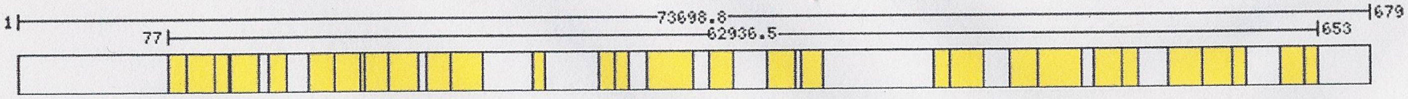


| Sort by: | Sequence | Position |
|---|---|---|
| PEPSTAT | AQFEGIVTDLIK | 349 - 360 |
| PEPSTAT | ASNGDAWVEAHGK | 147 - 159 |
| PEPSTAT | CELSSSVQTDINLPYLTMDASGPK | 317 - 340 |
| PEPSTAT | DAGQISGLNVLR | 207 - 218 |
| PEPSTAT | DDIENMVK | 556 - 563 |
| PEPSTAT | DNMALQR | 301 - 307 |
| PEPSTAT | EQQIVIQSSGGLSK | 542 - 555 |
| PEPSTAT | ETAENYLGHTAK | 176 - 187 |
| | | 293 - 300 |

# Sequest Summary

File Select Sequest Protein Utilities Help

**Sample:** Berne, M. (K301) 1 mb
**DataFiles:** K301 (04/05/04-04/05/04)
**Database:** rat (01/05/2004)
**Directory:** mbernek301 View Info
**Diff Mods:** 16 M 57.02 C 0.000 X

**OutFiles:** 1319|895
**Enzyme:** Trypsin_Strict
**Mass:** ±1.5 (Mono)
**Max list:** 1000
**Intensity:** 9.4e9 MS2 DTA VCR

Consensus **Depth:** 3

A gi|6729936|pdb|1MAB|G +1 76 16|Σ:7.1e8|8%|avg:4.5e7 {13,3,0,0,0,0}
Chain G, Rat Liver F1-Atpase gi|310190|gb|AAA41776.1| ATP synthase gamma-subunit [Rattus norvegicus] [MASS=29933] [MASS=29933]

| # | GBU | TIC | File | z | dM | MH+ | xC | dCn | Sp | RSp | Ions | Sf | Ref | () | Sequence | Prob |
|---|---|---|---|---|---|---|---|---|---|---|---|---|---|---|---|---|
| 1025 | | 1.4e8 | 3383-3402 | 2 | -2.2 | 3651.3 | 4.82 | 0.55 | 1181 | 1 | 22/64 | -- | gi\|6729936 | +1 | (-)KPPTFGDASVIALELLNSGYEFDEGSIIFNQFK | |
| 1021 | | 3.3e8 | 3368-3416 | 2 | 1.2 | 3647.9 | 4.50 | 0.57 | 1066 | 1 | 23/64 | -- | gi\|6729936 | +1 | (-)KPPTFGDASVIALELLNSGYEFDEGSIIFNQFK | |
| 683 | | 5.2e6 | 2149-2168 | 2 | -0.5 | 1755.4 | 3.27 | 0.48 | 677 | 1 | 17/28 | -- | gi\|6729936 | +1 | (-)NASDM*IDKLTLTFNR | |
| 557 | | 4.5e7 | 1972-2008 | 2 | 0.0 | 1309.4 | 3.16 | 0.53 | 741 | 1 | 16/20 | -- | gi\|6729936 | +1 | (-)THSDQFLVSFK | |
| 291 | | 8.3e7 | 1123-1170 | 2 | 0.2 | 1078.4 | 3.11 | 0.39 | 1262 | 1 | 17/20 | -- | gi\|6729936 | +1 | (-)NDM*AALTAAGK | |
| 563 | | 4.5e6 | 1980-2002 | 3 | -0.7 | 1310.2 | 3.09 | 0.27 | 568 | 2 | 21/40 | -- | gi\|6729936 | +1 | (-)THSDQFLVSFK | |
| 1050 | | 3.1e6 | 3480-3495 | 2 | -0.4 | 3649.5 | 3.02 | 0.58 | 473 | 1 | 17/64 | -- | gi\|6729936 | +1 | (-)KPPTFGDASVIALELLNSGYEFDEGSIIFNQFK | |
| 1034 | | 1.0e7 | 3431-3465 | 2 | -2.3 | 3651.4 | 3.00 | 0.41 | 276 | 1 | 13/64 | -- | gi\|6729936 | +1 | (-)KPPTFGDASVIALELLNSGYEFDEGSIIFNQFK | |
| 568 | | 1.4e6 | 1988-1993 | 1 | 1.1 | 1308.4 | 2.26 | 0.43 | 627 | 1 | 13/20 | -- | gi\|6729936 | +1 | (-)THSDQFLVSFK | |
| 802 | | 9.1e6 | 2480-2531 | 1 | 0.6 | 1314.9 | 2.25 | 0.25 | 245 | 10 | 9/24 | -- | gi\|6729936 | +1 | (-)ELIEIISGAAALD | |
| 462 | | 4.5e7 | 1752-1781 | 2 | -0.7 | 1091.3 | 2.05 | 0.29 | 454 | 1 | 13/18 | -- | gi\|6729936 | +1 | (-)EVM*IVGIGEK | |
| 502 | | 1.5e7 | 1847-1867 | 2 | 0.1 | 865.0 | 1.83 | ---- | 303 | 3 | 9/12 | -- | gi\|6729936 | +1 | (-)LTLTFNR | |
| 467 | | 4.5e6 | 1759-1777 | 1 | 0.1 | 1090.5 | 1.84 | 0.27 | 142 | 1 | 12/18 | -- | gi\|6729936 | +1 | (-)EVM*IVGIGEK | |
| 803 | | 2.6e6 | 2484-2513 | 1 | -1.0 | 1316.5 | 1.56 | 0.17 | 293 | 2 | 10/24 | -- | gi\|6729936 | +1 | (-)ELIEIISGAAALD | |
| 804 | | 5.2e5 | 2489-2507 | 2 | 0.7 | 1314.8 | 1.46 | ---- | 261 | 12 | 11/24 | -- | gi\|6729936 | +1 | (-)ELIEIISGAAALD | |
| 306 | | 1.1e7 | 1225-1249 | 1 | 0.5 | 696.3 | 0.94 | ---- | 207 | 1 | 6/10 | -- | gi\|6729936 | +2 | (-)SVISYK | |

B gi|27700611|ref|XP_230019.1| 36 4|Σ:1.2e6|0%|avg:3.1e5 {3,0,1,0,1,6}
titin [Rattus norvegicus] [MASS=1297407] [MASS=1297407]

| # | GBU | TIC | File | z | dM | MH+ | xC | dCn | Sp | RSp | Ions | Sf | Ref | () | Sequence | Prob |
|---|---|---|---|---|---|---|---|---|---|---|---|---|---|---|---|---|
| 30 | | 3.5e5 | 0253-0282 | 3 | -0.4 | 1415.9 | 1.37 | 0.15 | 154 | 50 | 10/44 | -- | gi\|27700611 | | (-)VDM*NDAGNFTC#R | |
| 181 | | 4.5e5 | 0460 | 2 | -1.5 | 875.5 | 1.09 | 0.03 | 153 | 50 | 6/14 | -- | gi\|27700611 | | (-)NTINKAGR | |
| 47 | | 2.5e5 | 0283 | 2 | -0.9 | 934.4 | 1.06 | 0.01 | 107 | 27 | 5/14 | -- | gi\|27700611 | | (-)IGDSIC#LR | |
| 1133 | | 1.8e5 | 3730 | 2 | 1.1 | 3250.7 | 0.92 | ---- | 96 | 27 | 9/56 | -- | gi\|27700611 | +1 | (-)YVCQIKNDAGMRECSALLTVLEPARIVEK | |

C gi|27666086|ref|XP_222242.1| 30 3|Σ:1.9e7|0%|avg:6.3e6 {3,0,0,0,0,0}
similar to mKIAA0219 protein [Mus musculus] [Rattus norvegicus] [MASS=313138] [MASS=313138]

| # | GBU | TIC | File | z | dM | MH+ | xC | dCn | Sp | RSp | Ions | Sf | Ref | () | Sequence | Prob |
|---|---|---|---|---|---|---|---|---|---|---|---|---|---|---|---|---|
| 951 | | 9.6e6 | 3128-3138 | 2 | 0.9 | 2782.3 | 1.72 | 0.03 | 51 | 376 | 7/50 | -- | gi\|27666086 | | (-)TVLNSHKVLPLETLVTDAGEVMETGK | |
| 567 | | 9.1e6 | 1987-2006 | 3 | -1.2 | 2020.2 | 1.72 | 0.08 | 133 | 265 | 14/60 | -- | gi\|27666086 | | (-)EM*LQAQMDKEAQIRRR | |
| 1210 | | 1.4e5 | 3935 | 2 | -1.1 | 2811.6 | 0.93 | 0.08 | 99 | 12 | 8/48 | -- | gi\|27666086 | | (-)RLQELDGELEAALGLLDAIM*TRNPR | |

D gi|17380501|sp|P16086|SPCN_RAT +1 42 5|Σ:3.8e7|0%|avg:7.5e6 {2,2,1,0,1,0}
Spectrin alpha chain, brain (Spectrin, non-erythroid alpha chain) (Alpha-II spectrin) (Fodrin alpha chain) gi|3462887|gb|AAC33127.1| alpha-fodrin [Rattus norvegicus] [MASS=284637] [MASS=284637]

| # | GBU | TIC | File | z | dM | MH+ | xC | dCn | Sp | RSp | Ions | Sf | Ref | () | Sequence | Prob |
|---|---|---|---|---|---|---|---|---|---|---|---|---|---|---|---|---|
| 543 | | 1.8e7 | 1949-1970 | 2 | 1.3 | 1524.3 | 2.26 | ---- | 407 | 12 | 11/22 | -- | gi\|17380501 | +4 | (-)DMDDEESWIKEK | |

# Flicka

Setup | CreateDTA | VuDTA | RunSequest | Status | Summary | Home

---

**Sample:** Berne, M. K301 1

**Send to:** PEPCUT PEPSTAT BLAST **NCBI:** SEQUENCE ABSTRACT

>**gi|6729936**|pdb|1MAB|G Chain G, Rat Liver F1-Atpase gi|310190|gb|AAA41776.1| ATP synthase gamma-subunit [Rattus norvegicus] [MASS=29933] [MASS=29933]
RDITRRLKSI KNIQKITKSM KMVAAAKYAR AERELKPARV YGTGSLALYE KAEIKGPEDK KKHLIIGVSS DRGLCGAIHS
SVAKQMK**NDM AALTAAGKEV MIVGIGEK**IK SILYR**THSDQ FLVSFK**DVGR **KPPTFGDASV IALELLNSGY EFDEGSIIFN**
**QFKSVISYK**T EEKPIFSFST VVAAENMSIY DDIDADVLQN YQEYNLANII YYSLKESTTS EQSARMTAMD NASK**NASDMI**
**DKLTLTFNR**T RQAVITK**ELI EIISGAAALD**

**Mass (mono):** 29914.6 **Identifier:** gi|6729936 **Database:** C:/database/rat.fasta
**Protein Coverage:** 99/270 = **36.7%** by amino acid count, 10727.5/29914.6 = **35.9%** by mass

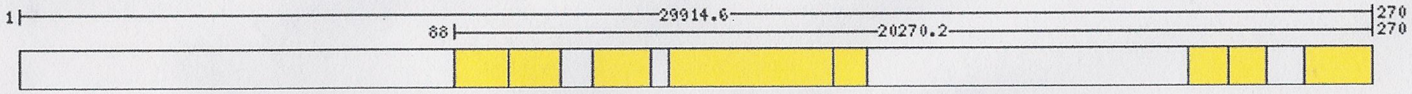


**Sort by:** **Sequence** ☐ **Position** ☐

| | Sequence | Position |
|---|---|---|
| PEPSTAT | ELIEIISGAAALD | 258 - 270 |
| PEPSTAT | EVMIVGIGEK | 99 - 108 |
| PEPSTAT | KPPTFGDASVIALELLNSGYEFDEGSIIFNQFK | 131 - 163 |
| PEPSTAT | LTLTFNR | 243 - 249 |
| PEPSTAT | NASDMIDKLTLTFNR | 235 - 249 |
| PEPSTAT | NDMAALTAAGK | 88 - 98 |
| PEPSTAT | SVISYK | 164 - 169 |
| PEPSTAT | THSDQFLVSFK | 116 - 126 |
