# Supplementary figures and images for: Mitochondrial impairment in the five-sixth nephrectomy model of chronic renal failure: proteomic approach
Source: BMC Nephrol. 2013 Oct 4;14:209. doi: 10.1186/1471-2369-14-209 (PMC3851543; doi:10.1186/1471-2369-14-209)

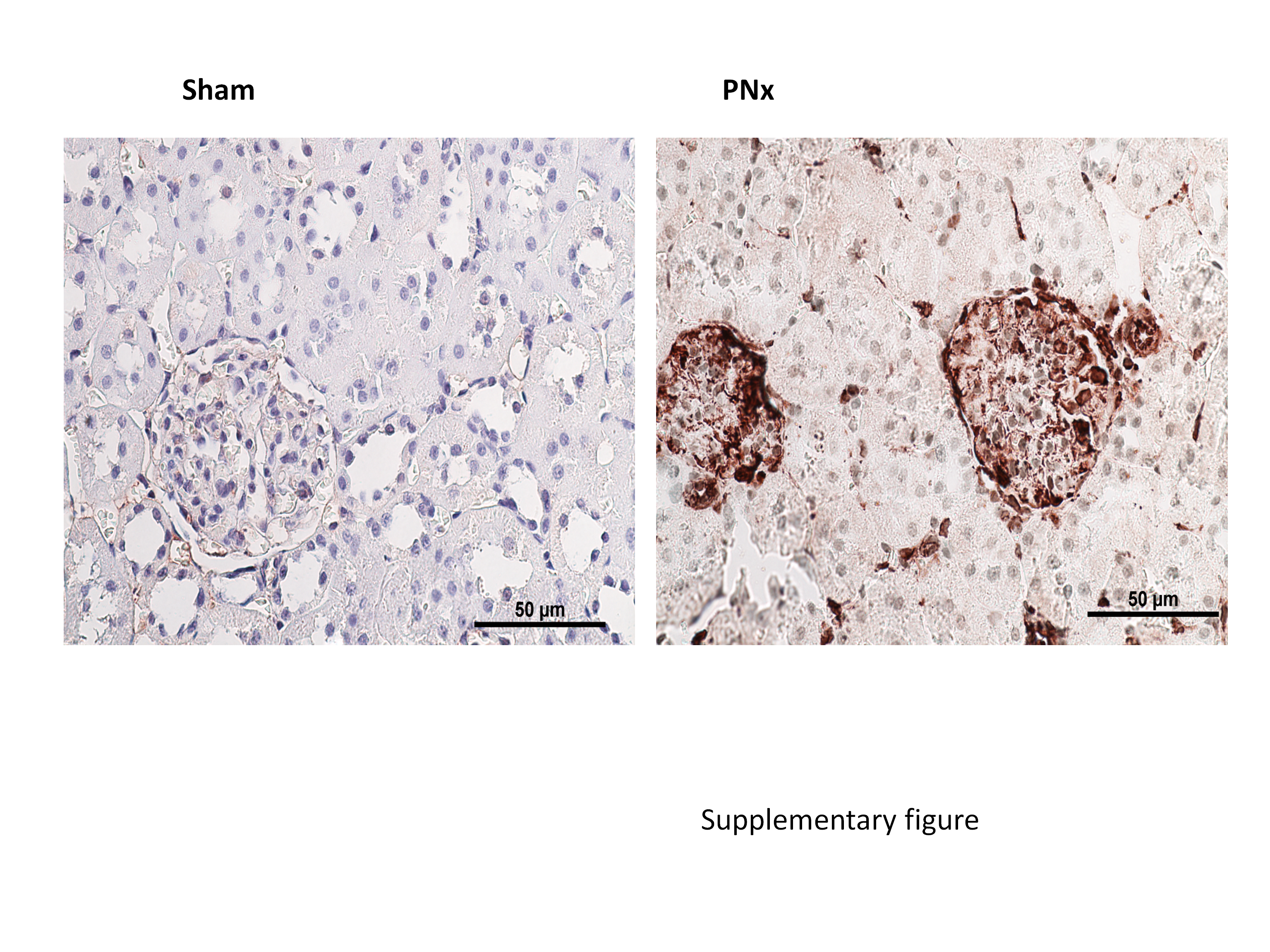

Supplement: Additional file 2: Figure S1 — Immunohistochemical analysis of vimentin protein in the cortex of sham-operated and PNx rats. [file 1471-2369-14-209-S2.tiff]

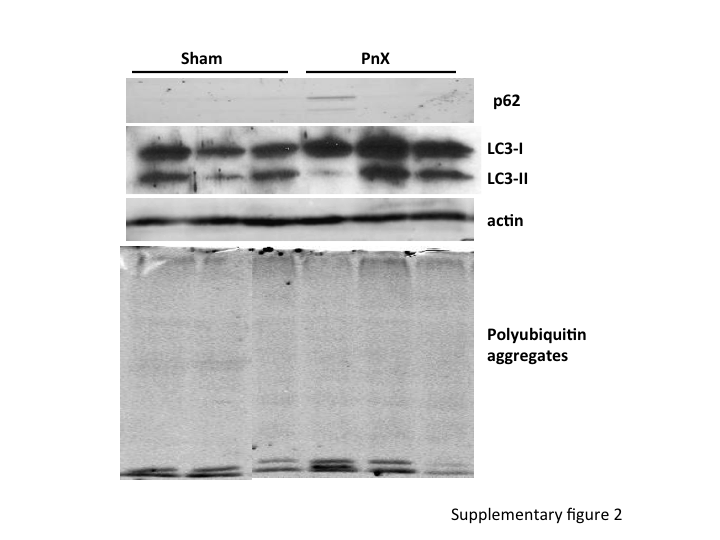

Supplement: Additional file 3: Figure S2 — Representative immunoblot of renal cortical extracts shows accumulation of p62 and very low level of LC3-II in only one PNx rat. There was slight difference in amounts of polyubuquinated proteins in cortex of the same PNx rat. [file 1471-2369-14-209-S3.tiff]
